# Highly Substituted Benzophenone Aldehydes and Eremophilane Derivatives from the Deep-Sea Derived Fungus *Phomopsis lithocarpus* FS508

**DOI:** 10.3390/md16090329

**Published:** 2018-09-11

**Authors:** Jian-Lin Xu, Hong-Xin Liu, Yu-Chan Chen, Hai-Bo Tan, Heng Guo, Li-Qiong Xu, Sai-Ni Li, Zi-Lei Huang, Hao-Hua Li, Xiao-Xia Gao, Wei-Min Zhang

**Affiliations:** 1 State Key Laboratory of Applied Microbiology Southern China, Guangdong Provincial Key Laboratory of Microbial Culture Collection and Application, Guangdong Open Laboratory of Applied Microbiology, Guangdong Institute of Microbiology, Guangzhou 510070, China; 15876503402@163.com (J.-L.X.); liuhx@gdim.cn (H.-X.L.); chenyc@gdim.cn (Y.-C.C.); Hengguo163@163.com (H.G.); xlq9513@126.com (L.-Q.X.); maibao66@126.com (S.-N.L.); huangzilei15@mails.ucas.ac.cn (Z.-L.H.); lihh@gdim.cn (H.-H.L.); 2 College of Pharmacy, Guangdong Pharmaceutical University, Guangzhou 510006, China; 3 Program for Natural Products Chemical Biology, Key Laboratory of Plant Resources Conservation and Sustainable Utilization, Guangdong Provincial Key Laboratory of Applied Botany, South China Botanical Garden, Chinese Academy of Sciences, Guangzhou 510650, China; tanhaibo@scbg.ac.cn

**Keywords:** deep-sea derived fungus, *Phomopsis lithocarpus*, benzophenone derivatives, eremophilane derivative

## Abstract

Five new benzophenone derivatives named tenellones D–H (**1**–**5**), sharing a rare naturally occurring aldehyde functionality in this family, and a new eremophilane derivative named lithocarin A (**7**), together with two known compounds (**6** and **8**), were isolated from the deep marine sediment-derived fungus *Phomopsis lithocarpus* FS508. All of the structures for these new compounds were fully characterized and established on the basis of extensive spectroscopic interpretation and X-ray crystallographic analysis. Compound **5** exhibited cytotoxic activity against HepG-2 and A549 cell lines with IC_50_ values of 16.0 and 17.6 μM, respectively.

## 1. Introduction

Fungi isolated from the deep-sea sediment are one of the most pivotal and promising source for bioactive compounds, presumably owing to the chemical diversity and biodiversity of their secondary metabolites that could be used for drug discovery and pharmacological applications [1,2,3]. In contrast to the terrestrial fungi, the majority of marine counterparts remain underexplored. Until now, a large number of natural products with intriguing structural skeleton and promising pharmacological effect have been discovered from marine-derived fungi, making them one of the attractive repositories for therapeutic agents and lead compounds [4,5,6]. Fingolimod, a synthetic derivative of immunosuppressive myriocin obtained from marine-derived *Myriococcum albomyces*, has been approved by the Food and Drug Administration and applied in patients with multiple sclerosis. Plinabulin, a synthetic analogue of the diketopiperazine halimide isolated from marine-derived *Aspergillus* sp., leading to disruption of microtubules and apoptosis, is currently examined in Phase III clinical trials for the treatment of non-small cell lung cancer. These findings have inspired natural product researchers to concentrate on the new marine compounds with pharmaceutical potential [7,8,9,10,11,12]. Fungi of the genus *Phomopsis*, widely distributed in both terrestrial and marine environments, are capable of producing structurally fascinating and architecturally diverse natural products, such as steroids [13], cytochalasins [14], pyrenocines [15], sesquiterpenes [16], diterpenes [17], terpenoids [18], oblongolides [19], which not only displayed multiple bioactivities including anti-inflammatory, antifungal, antibacterial, antiviral, antimigratory activities as well as cytotoxicity, but also provided several classes of new scaffolds that could be further modified to obtain the desired pharmaceutical effect. Accordingly, it is worthwhile to make efforts for the discovery of novel bioactive metabolites from untapped species of the genus *Phomopsis*.

**Figure 1:**
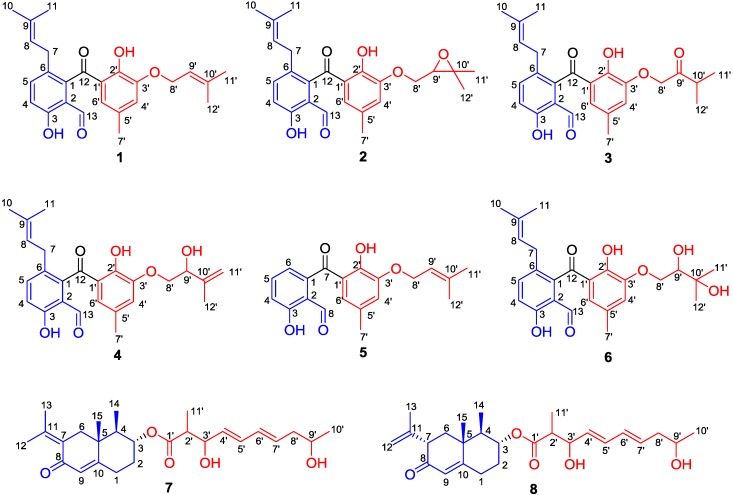
The structures of compounds **1**–**8**.

Motivated by an ongoing research program focusing on biologically meaningful natural products with novel structural diversity and architectural complexity from the marine-derived fungi, numerous new compounds with cytotoxic and enzyme inhibitory activities were obtained, including lithocarpins, dichotocejpins, eutypellols and acaromycin [20,21,22,23,24]. Recently, a continuing investigation of *Phomopsis lithocarpus* FS508, which was isolated from the sediment sample of the India Ocean, led to obtain five new benzophenone derivatives with a rare naturally occurring aldehyde functionality named tenellones D–H (**1**–**5**) as well as a new eremophilane derivative lithocarin A (**7**), together with two related known compounds tenellone A [25] and AA03390 [26] (Figure 1). The structures of all the isolates were fully characterized and rationally established by comprehensive spectroscopic analyses and X-ray crystallography. All of the compounds were evaluated for their antitumor activity, and compound **5** displayed moderate inhibitory effect against human tumor cell lines HepG-2 and A549 with IC_50_ values of 16.0 and 17.6 μM, respectively.

## 2. Results and Discussion

### 2.1. Structure Elucidation

Tenellone D (**1**) was obtained as yellow needles. Its molecular formula was determined as C_25_H_28_O_5_ on the basis of negative HRESIMS with a deprotonated molecular ion at *m*/*z* 407.1867 [M − H]^−^ (calcd. for C_25_H_27_O_5_, 407.1864), indicating the existence of 12 indices of hydrogen deficiency. The IR spectrum of **1** showed two unambiguous absorption bands at 3445 cm^−1^ and 1653 cm^−1^, which were characteristic for hydroxy and carbonyl functionalities, respectively. With a careful inspection and analyses of its ^13^C NMR (Table 1) and HSQC spectra (Figure S5), 25 carbon signals were successfully distinguished and ascribed to two carbonyl moieties, five methyls, two methylenes, six methines, as well as ten quaternary carbons.

**Figure 2:**
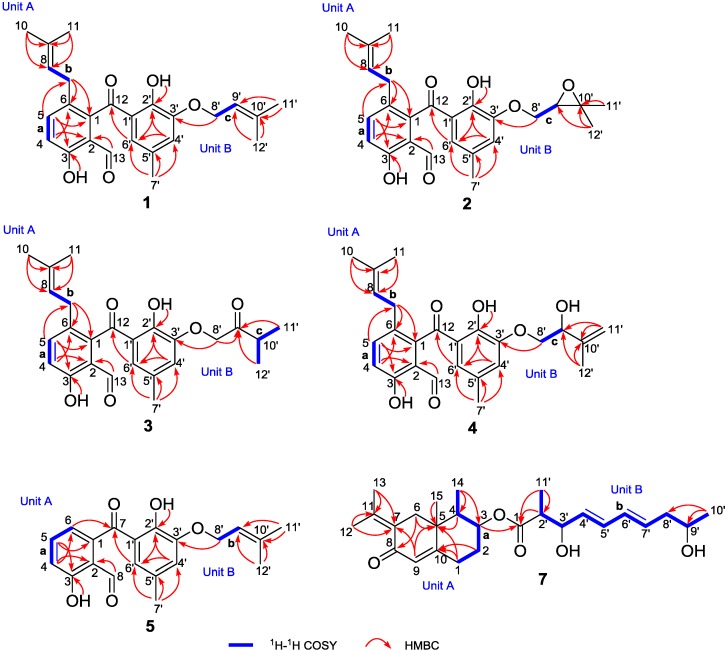
^1^H-^1^H COSYs and key HMBCs of **1**–**5**, and **7**.

The proton–proton connectivities of **1** clearly clarified the existence of three spin-coupling systems: **a** (H-4/H-5), **b** (H-7/H-8), and **c** (H-8’/H-9’) as depicted in Figure 2. On the basis of fragment **a**, a 1,2,3,6-tetrasubstituted aromatic ring was initially established attributable to the critical HMBC correlations from H-4 to C-2 and C-6 as well as H-5 to C-1 and C-3. Moreover, the HMBC correlations from H-10 and H-11 to C-9 and C-8, coupled with the fragment **b**, strongly suggested the presence of an isopentenyl fragment; and it was concluded to link at C-6 (*δ*_C_ 129.9) position in the 1,2,3,6-tetrasubstituted aromatic ring, which could be further evidenced by pivotal HMBC interactions from the methylene protons H-7 (*δ*_H_ 3.12) to C-1 and C-6. Similarly, the location of the aldehyde group C-13 (*δ*_C_ 194.5) at C-2 (*δ*_C_ 117.4) in the aromatic ring was reasonably verified by the conclusive HMBC correlations from the aldehyde proton H-13 (*δ*_H_ 9.71) to C-2 and C-3. The position of hydroxyl group was deduced to connect at C-3 mainly referred to its significant down-shifted carbon signal at *δ*_C_ 160.8, which could be further supported by the HMBC correlations from hydroxyl proton (*δ*_H_ 11.51) to C-3 and C-4. Therefore, the unit A was finally established as shown in Figure 2.

In unit B, the second 1’,2’,3’,5’-tetrasubstituted benzene ring with a phenolic group at C-2’ (*δ*_C_ 151.7) was readily constructed by the *meta*-coupled aromatic protons of H-4’ (*δ*_H_ 6.93) and H-6’ (*δ*_H_ 6.47), the aforementioned conclusion could be further confirmed and supported by the HMBC interactions of H-4’/C-2’, H-4’/C-6’, as well as H-6’/C-2’. In addition, the HMBC correlations from H-7’ (*δ*_H_ 2.17) to C-4’, C-5’, and C-6’ strongly suggested the location of the methyl group at C-5’ (*δ*_C_ 128.6). Furthermore, on the basis of fragment **c**, the HMBC interactions from H_3_-11’ and H_3_-12’ to C-9’ as well as H_3_-11’ and H_3_-12’ to C-10’ implied the presence of another isoprenyl moiety in unit B. This isoprenyl group could be readily assigned at C-3’ attributable to the pivotal HMBC correlation of the methylene protons H-8’ (*δ*_H_ 4.62) to C-3’ (*δ*_C_ 148.0). Therefore, the unit B was completely ascertained.

**Figure 3:**
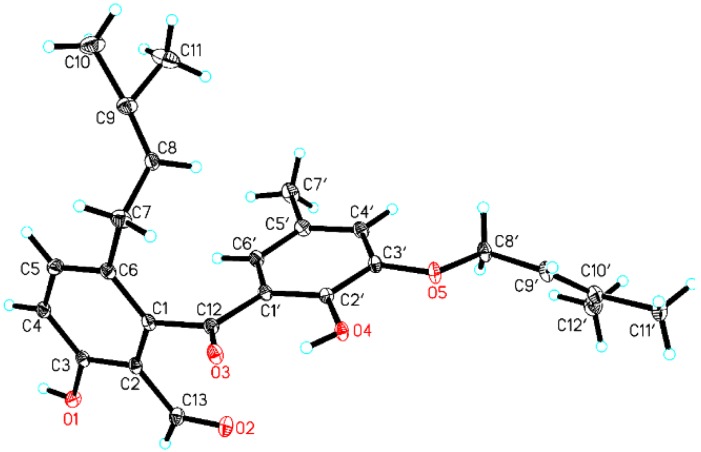
Perspective drawing of the X-ray structure of **1**.

The connectivity of the units A and B was initially speculated to conjunct through a carbonyl carbon atom C-12 (*δ*_C_ 203.1) with the formation of a benzophenone architecture, which was mainly attributed to the HMBC correlation between H-6’ and C-12. However, the linkage of the unit A and the ketone group was still a critical uncertainty for the elucidation of the structure of **1** because of the lack of any conclusive long-range correlations. Fortunately, after many attempts with different solvent combinations, a single crystal of compound **1** suitable for X-ray crystallographic analysis was obtained. The further X-ray crystallographic measurement was conducted and completed on the Cu K*α*, which adequately clarified the structure of **1** without ambiguity (Figure 3). In light of the aforementioned evidence, the structure of **1** was concluded as shown in Figure 2.

Compound **2** was isolated as a yellow oil. The molecular formula was assigned as C_25_H_28_O_6_ based on the negative mode HRESIMS [M + Cl]^−^ ion at *m*/*z* 459.1583 (calcd. for 459.1580), indicating the presence of twelve degrees of unsaturation in the molecule. The ^1^H and ^13^C NMR spectra of **2** closely resembled to those of **1** except for the presence of two oxygenated alkyl carbons (*δ*_C_ 71.1, 76.6) and the absence of two olefinic carbons in **2**, suggesting that the olefinic bond should be replaced by an epoxy moiety in **2** because of the slight up-shifted proton signal (*δ*_H_ 4.07) affiliated to the oxygenated alkyl carbon, which could be also accessibly confirmed by the ^1^H-^1^H COSY cross peak of H-8’/H-9’ and the HMBC correlations from H-11’ (*δ*_C_ 1.69) and H-12’ (*δ*_C_ 1.71) to C-10’ (*δ*_C_ 71.1) and C-9’ (*δ*_C_ 76.6). Thus, the structure of **2** was elucidated as shown in Figure 2 and given the trivial name tenellone E.

Compound **3** was purified as a yellow oil. Its molecular formula was assigned to be the same as that for compound **2** on the basis of their HRESIMS data. The 1D NMR data (Table 2) of **3** was similar to those of **2**, indicating that they should share a very similar tenellone skeleton. After a careful inspection and close comparison, the major differences were disclosed to be the presence of a ketone functionality (*δ*_C_ 210.6) and a more high-field carbon (*δ*_C_ 37.2) in **3** instead of the epoxy moiety in **2**. As referring to the HMBC spectrum, the obvious cross peaks of H-8’ to C-9’, H-11’ to C-9’, and H-12’ to C-9’ were successfully distinguished, and it unambiguously verified the existence of a 3-methylbutan-2-one fragment in **3**. Moreover, the ^1^H-^1^H COSY correlations of H-10’/H-11’ and H-10’/H-12’ further accounted for the above deduction. Therefore, compound **3** was completely determined (Figure 2) and given the trivial name of tenellone F.

Tenellone G (**4**) was obtained as a yellow oil, which also shared the same molecular formula C_25_H_28_O_6_ inferred from a HRESIMS with a pseudo-molecular peak at *m*/*z* 423.1825 ([M − H]^−^, calcd. for 423.1813). From the typical NMR data between compounds **1** and **4**, it could be constructively concluded that both of them should feature a similar tenellone structure, except for the absence of a methyl group (*δ*_C_ 26.0) in **1** and the presence of a oxygenated methine (*δ*_C_ 74.0) as well as a terminal olefin (*δ*_C_ 112.2, 146.0) in **4**. These differences were attributed to the oxidative rearrangement of the isoprenyl moiety, leading to an allylic alcohol with a disubstituted double bond. This deduction could be further rationalized by the critical HMBC correlations from H-11’ (*δ*_H_ 4.92, 5.12) and H-12’ (*δ*_H_ 1.83) to C-9’ (*δ*_C_ 74.0) and C-10’ (*δ*_C_ 146.0). Thereby, the structure of **4** was ascertained as depicted in Figure 2.

Tenellone H (**5**) was obtained as a yellow powder, and its molecular formula was assigned as C_20_H_20_O_5_ based on the negative mode HRESIMS with an obvious molecular ion peak at *m*/*z* 339.1243 ([M − H]^−^, calcd. for 339.1238. A detailed inspection and comparison of the 1D NMR spectra between **1** and **5** clearly revealed that both of them should possess the same structural architecture, which could be further confirmed by the identical proton-proton and carbon-proton correlations (Table 3). The notable differences of two compounds were ascribed to the absence of five carbons (*δ*_C_ 17.8, 25.7, 31.0, 121.9, 134.1) in **5**, which were characteristic for an isoprenyl group, suggesting the inexistence of the isoprenyl moiety attached at C-6 in aromatic ring A for compound **5**. Moreover, the predominant correlations from H-6 to C-4, C-7, and C-2 also strengthened this conclusion. Thus, compound **5** was finally determined as a de-isoprenyl derivative of compound **1** (Figure 2).

Compound **7** was isolated as a yellow oil. Its molecular formula was deduced as C_26_H_38_O_5_, which was determined on the basis of the HRESIMS peak in conjunction with ^13^C NMR data (Table 4), requiring eight indices of hydrogen deficiency. The ^1^H-^1^H COSY coupled with HSQC spectra initially disclosed two spin-coupling fragments as depicted with bold blue lines in Figure 2: **a** (H-1/H-2/H-3/H-4/H-14) and **b** (H-11’/H-2’/H-3’/H-4’/H-5’/H-6’/H-7’/H-8’/H-9’/H-10’). The HMBC correlations from H-1 to C-5 and C-10 as well as H-4 to C-5, coupled with the fragment **a**, successfully established the six-membered cyclic ring with the methyl group at C-4, which could be rationally verified by the HMBC correlations of H_3_-14 (*δ*_H_ 0.98) to C-3, C-4, and C-5.

Moreover, the HMBC showed long-range ^1^H-^13^C correlations from H-6 (*δ*_H_ 2.16 and 2.91) to C-5 and C-8 as well as H-9 (*δ*_H_ 5.77) to C-1, C-5, and C-7, revealing the presence of the 2-decalone substructure. The connection between methyl group at C-15 and 2-decalone moiety was deduced to be located at C-5, which was further secured by the critical HMBC interactions from H-15 to C-4, C-5, and C-6. Additionally, HMBC correlations of H-12 and H-13 to C-7 and C-11 adequately assigned the location of 2-propene at C-7. Thus, the unit A was finally determined (Figure 2). The HMBC correlations from H-2’/C-1’ and H-11’/C-1’ indicated the structural motif of 2-methyl-3,9-dihydroxydecenoic acid (C-1’-C-11’) with the aid of the fragment **b**. Consequently, the unit B was ascertained. The key cross peak of H-3 (*δ*_H_ 4.87) to C-1’ (*δ*_C_ 175.2) was observed, which unambiguously confirmed the linkage of units A and B through C-3 (*δ*_C_ 74.2). Therefore, the planar structure of **7** was determined and given the trivial name lithocarin A.

**Figure 4:**
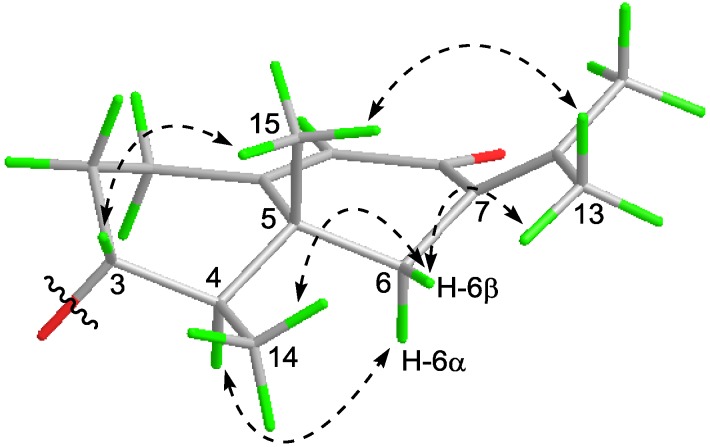
Key NOESY correlations of compound **7** (sesquiterpenoid moiety).

The relative configuration of **7** was clarified on the basis of the coupling constant data and NOESY spectrum (Figure 4). The observed obvious NOESY correlation of H-3/H_3_-15 suggested that H-3 and H_3_-15 were co-facial and arbitrarily assigned to be in β-orientation. The large ^3^*J*_H-3,H-4_ (11.2 Hz) indicated the *trans*-relationship between H-3 and H-4, assigning the H-4 in α-orientation. Meanwhile, NOESY correlation from H-4 to H-6α (δ_H_ 2.16) implied that H-6α was α-orientation. Then, the methyl group at C-4 was β-orientation because of the NOESY correlation between H_3_-14 to H-6β*.* The geometries of the double bonds (C-4’ and C-6’) could be rationally determined as 4’*E* and 6’*E* evidenced by the identical coupling constants of H-4’ and H-5’ as well as H-6’ and H-7’ (*J* = 15.0 Hz). 

### 2.2. Cytotoxicity Assay

Compounds **1**–**8** were evaluated for their cytotoxic activities against the HepG-2, MCF-7, SF-268, and A549 tumor cell lines with cisplatin as the positive control. Compound **5** exhibited moderate inhibitory activity against HepG-2 and A549 cell lines with IC_50_ values of 16.0 and 17.6 μM, respectively; while compound **8** showed weak inhibitory effect against the tested tumor cell lines with the IC_50_ values ranging from 25.5 to 29.6 μM. The other six compounds exhibited no cytotoxic activity even at the concentration of 50 µM (Table 5). 

## 3. Materials and Methods 

### 3.1. General Experimental Procedures

UV spectra were taken on a Shimadzu UV-2600 spectrophotometer (Shimadzu, Kyoto, Japan). IR data were recorded on a Shimadzu IR Affinity-1 spectrometer (Shimadzu, Kyoto, Japan). Optical rotations were measured on an Anton Paar MCP-500 spectropolarimeter (Anton Paar, Graz, Austria) at 25 °C. Circular dichroism (CD) spectra were obtained under N_2_ gas on a Jasco 820 spectropolarimeter (Jasco Corporation, Kyoto, Japan). The NMR spectra were acquired using a Bruker Avance 500 MHz or 600 MHz NMR spectrometer with TMS as an internal standard (Bruker, Fällanden, Switzerland). ESIMS data were collected on an Agilent Technologies 1290-6430A Triple Quad LC/MS (Agilent Technologies, Palo Alto, CA, USA). HRESIMS were done with a Thermo MAT95XP high resolution mass spectrometer (Thermo Fisher Scientific, Bremen, Germany). Preparative HPLC separations were carried out using a YMC-pack ODS-A column (250 × 20 mm, 5 μm, 12 nm, YMC Co., Ltd, Kyoto, Japan). Semi-preparative HPLC separations were performed utilizing a YMC-pack ODS-A/AQ column (250 × 10 mm, 5 μm, 12 nm, YMC CO., Ltd, Kyoto, Japan) and a YMC-pack Cellulose-SB column (250 × 10 mm, 5 μm, 12 nm, YMC CO., Ltd, Kyoto, Japan). Column chromatography were performed with silica gel (200−300 mesh, Qingdao Marine Chemical Inc., Qingdao, China) and Sephadex LH-20 (Amersham Biosciences, Uppsala, Sweden), respectively. Thin-Layer Chromatography (TLC) was conducted with precoated glass plates GF-254 (Merck KGaA, Darmstadt, Germany).

### 3.2. Fungal Material

The fungus *P. lithocarpus* FS508 was isolated in 2016 from a deep-sea sediment sample collected in the Indian Ocean (111°53.335’ E, 16°50.508’ N; depth 3606 m). The sequence of amplified ITS region of the strain FS508 has been submitted to GenBank (Accession No. MG686131). A BLAST search of ITS region revealed that FS508 has 99% homology with *Phomopsis lithocarpus* CZ105B (Accession No. FJ755236). The strain is preserved at the Guangdong Provincial Key Laboratory of Microbial Culture Collection and Application, Guangdong Institute of Microbiology.

### 3.3. Fermentation, Extraction and Isolation

The fermentation was carried out in 3 L Erlenmeyer flasks, which contained 480 g of rice and 600 mL of 0.5% saline water. Each flask was aseptically inoculated with the seed inoculums and statically fermented for a month at 28 °C. The fermented rice substrate (10 flasks) was extracted three times with EtOAc, and the solvent was evaporated to dryness under vacuum to obtain a crude extract (99.8 g). The crude extract was completely dissolved in 80% MeOH/H_2_O, which was extracted with petroleum ether four times to remove some aliphatic acids. The obtained residue was subjected to silica gel chromatography (200−300 mesh) by step gradient elution with petroleum ether/EtOAc (10:1→0:1) and followed by CH_2_Cl_2_/MeOH in linear gradient (8:1→0:1) to yield 14 fractions (Frs. 1−14). 

Fr. 4 (0.22 g) was further fractionated by column chromatography on silica gel eluting with a *n*-hexane/EtOAc (6:1→1:1) to produce 3 fractions. Fr. 4-1 (0.06 g) was re-purified by HPLC on a semipreparative YMC-pack ODS-A/AQ column (MeOH/H_2_O, 85:15, 3 mL/min) to obtain **1** (3.0 mg, t*_R_* 21.8 min) and **5** (1.3 mg, t*_R_* 12.1 min). Fr. 5 (0.36 g) was separated by preparative RP-HPLC system over a YMC ODS-A column (MeOH/H_2_O, 90:10, 8 mL/min) to afford 3 fractions. Fr. 5-1 (0.08 g) was further fractionated on a semipreparative YMC-pack Cellulose-SB column (MeOH/H_2_O, 82:18, 3 mL/min) to give 2 subfractions. Fr. 5-1-1 was purified by HPLC on a semipreparative YMC-pack ODS-A/AQ column (MeOH/H_2_O, 86:14, 3 mL/min) to obtain **2** (3.2 mg, t*_R_* 10.7 min) and **3** (1.2 mg, t*_R_* 10.1 min). Fr. 6 (3.10 g) was fractionated by Sephadex LH-20, eluting with CH_2_Cl_2_/MeOH (1:1) to yield 4 fractions. Fr. 6-2 (2.20 g) was separated into 5 fractions on a silica gel column (200−300 mesh), eluting with petroleum ether/EtOAc in linear gradient (6:1→0:1). Fr. 6-2-1 (1.20 g) was re-purified by preparative RP-HPLC system over a YMC ODS-A column (MeOH/H_2_O, 85:15, 8 mL/min) to produce 5 subfractions. Fr. 6-2-1-3 was further purified by HPLC on a semipreparative YMC-pack ODS-A/AQ column (MeCN/H_2_O, 82:18, 3 mL/min) to obtain **4** (4.2 mg, t*_R_* 9.8 min). Fr. 8 (15.00 g) was separated by column chromatography over C-18 reversed-phase (RP) silica gel eluting with a MeOH/H_2_O gradient (30:70→100:0) to produce 13 fractions. Fr. 8-12 (1.30 g) was subjected to a silica gel column (200-300 mesh) using a stepped gradient elution of petroleum ether/EtOAc (6:1→0:1) to give 3 fractions. Fr. 8-12-1 (0.06 g) was further purified by HPLC on a semipreparative YMC-pack ODS-A/AQ column (MeOH/H_2_O, 80:20, 3 mL/min) to obtain **6** (2.1 mg, t*_R_* 12.6 min). Fr. 8-12-2 (0.36 g) was separated over Sephadex LH-20 into 4 subfractions, eluting with CH_2_Cl_2_/MeOH (1:1). Fr. 8-12-2-1 (0.07 g) was re-purified by HPLC on a semipreparative YMC-pack ODS-A/AQ column (MeOH/H_2_O, 77:23, 3 mL/min) to afford **7** (3.9 mg, t*_R_* 15.7 min) and **8** (10.0 mg, t*_R_* 14.5 min).

Tenellone D (**1**): yellow needles; UV (MeOH) *λ*_max_ (log *ε*) 218.2 (4.12), 266.6 (3.60), 346.2 (3.26) nm; IR *ν*_max_ 3445, 2924, 1653, 1456, 1261 cm^−1^. ^1^H (600 MHz) and ^13^C (150 MHz) NMR spectral data, see Table 1; negative ESIMS: *m*/*z* 407 [M − H]^–^; HRESIMS: *m*/*z* 407.1867 [M − H]^–^ (calcd. for C_25_H_27_O_5_, 407.1864).

Tenellone E (**2**): yellow oil; [α]${}_{D}^{25}$ +10.3 (*c* 0.99, MeOH). UV (MeOH) *λ*_max_ (log *ε*) 218.6 (4.60), 266.0 (4.11), 346.2 (3.87) nm; IR *ν*_max_ 3502, 2926, 1653, 1456, 1265 cm^−1^. ^1^H (600 MHz) and ^13^C (150 MHz) NMR spectral data, see Table 1; negative ESIMS: *m*/*z* 459 [M + Cl]^−^; HRESIMS: *m*/*z* 459.1583 [M + Cl]^−^ (calcd. for C_25_H_28_O_6_Cl, 459.1580).

Tenellone F (**3**): yellow oil; UV (MeOH) *λ*_max_ (log *ε*) 216.0 (4.40), 267.4 (3.92), 345.4 (3.63) nm; IR *ν*_max_ 3377, 2924, 1651, 1456, 1267 cm^−1^. ^1^H (600 MHz) and ^13^C (150 MHz) NMR spectral data, see Table 2; negative ESIMS: *m*/*z* 423 [M − H]^–^; HRESIMS: *m*/*z* 423.1808 [M − H]^–^ (calcd. for C_25_H_27_O_6_, 423.1813).

Tenellone G (**4**): yellow oil; [α]${}_{D}^{25}$ +10.0 (*c* 1.02, MeOH). UV (MeOH) *λ*_max_ (log *ε*) 218.6 (4.61), 266.0 (4.12), 346.0 (3.88) nm; IR *ν*_max_ 3356, 2926, 1647, 1450, 1273 cm^−1^. ^1^H (600 MHz) and ^13^C (150 MHz) NMR spectral data, see Table 2; negative ESIMS: *m*/*z* 423 [M − H]^–^; HRESIMS: *m*/*z* 423.1825 [M − H]^–^ (calcd. for C_25_H_27_O_6_, 423.1813).

Tenellone H (**5**): yellow powder; UV (MeOH) *λ*_max_ (log *ε*) 213.0 (4.58), 265.8 (4.06), 335.4 (3.75) nm; IR *ν*_max_ 3377, 2931, 1651, 1474, 1022 cm^−1^. ^1^H (600 MHz) and ^13^C (150 MHz) NMR spectral data, see Table 3; negative ESIMS: *m*/*z* 339 [M − H]^–^; HRESIMS: *m*/*z* 339.1243 [M − H]^–^ (calcd. for C_20_H_20_O_5_, 339.1238).

Lithocarin A (**7**): yellow oil; [α]${}_{D}^{25}$ −16.3 (*c* 0.98, MeOH). UV (MeOH) *λ*_max_ (log *ε*) 204.0 (4.37), 235.2 (4.50), 276.0 (4.12) nm; CD (0.20 mg/mL, MeOH) *λ*_max_ (Δ*ε*) 215 (−11.40), 241 (+35.00), 282 (−16.67), 326 (−1.73) nm; IR *ν*_max_ 3366, 2939, 1717, 1655, 1373 cm^−1^. ^1^H (500 MHz) and ^13^C (125 MHz) NMR spectral data, see Table 4; positive ESIMS: *m*/*z* 453 [M + Na]^+^; HRESIMS: *m*/*z* 453.2609 [M + Na]^+^ (calcd. for C_26_H_38_O_5_, 453.2611).

### 3.4. X-Ray Analysis of Tenellone D (1)

The single-crystal X-ray diffraction data of compound **1** was collected at 100 K on Rigaku Oxford Diffraction Supernova Dual Source, Cu at Zero equipped with an AtlasS2 CCD using Cu K*α* radiation. Data reduction was carried out with the diffractometer’s software. The structures were solved by direct methods using Olex2 software, and the non-hydrogen atoms were located from the trial structure and then refined anisotropically with SHELXL-2014 using a full-matrix least squares procedure based on *F^2^*. The weighted *R* factor, *wR* and goodness-of-fit *S* values were obtained based on *F*^2^.The hydrogen atom positions were fixed geometrically at the calculated distances and allowed to ride on their parent atoms. Crystallographic data for the structure of tenellone D (**1**) reported in this paper has been deposited in the Cambridge Crystallographic Data Centre. (Deposition number: CCDC 1852781). Copies of these data can be obtained free of charge via www.ccdc.cam.au.ck/conts/retrieving.html.

Crystal data for compound **1**: C_25_H_28_O_5_, *M* = 408.47, monoclinic, size 0.13 × 0.12 × 0.11 mm^3^, space group P2_1_/c; *a* = 9.9962 (3) Å, *b* = 21.9296 (6) Å, *c* = 10.3159 (3) Å, α = 90.00°, β = 109.354(4), γ = 90.00°, *V* = 2133.57 (12) Å^3^, *T* = 100.00 K, *Z* = 4, ρ_calcd._ = 1.272 g/cm^3^, *F*(000) = 872.0, 8355 reflections in −12 ≤ *h* ≤ 6, −26 ≤ *k* ≤ 26, −12 ≤ *l* ≤ 12, measured in the range 8.064° ≤ 2θ ≤ 147.114°, GOOF = 1.036, Final *R* indices I > 2σ(I): *R*_1_ = 0.0494, w*R*_2_ = 0.1245, Final *R* indices (all data): *R*_1_ = 0.0579, w*R*_2_ = 0.1328, largest difference peak and hole = 0.27 and −0.24 e. Å^−3^.

### 3.5. Cytotoxicity Assay

The cytotoxic activities of compounds (**1**–**8**) were evaluated against four human tumor cell lines HepG-2, MCF-7, SF-268, and A549 with cisplatin as the positive control. Assays were performed by the Sulforhodamine (SRB) method [27].

## 4. Conclusions

In summary, five new benzophenone derivatives and a new eremophilane derivative, along with two known compounds, were isolated from the marine-derived fungus *P. lithocarpus* FS508. The chemical structures of eight compounds were elucidated by means of NMR analyses and single-crystal X-ray diffraction. The antitumor activities of compounds **1**–**8** were evaluated wherein compound **5** exhibited moderate growth inhibition against HepG-2 and A549 cell lines with IC_50_ values of 16.0 and 17.6 μM, respectively; while compound **8** displayed weak inhibitory effect against four human tumor cell lines with the IC_50_ values ranging from 25.5 to 29.6 μM. However, compounds **1**–**4**, **6** and **7** were inactive against these tumor cell lines even at 50 µM. Comparing with compound **5**, compounds **1**–**4** and **6**, possessing an isoprenyl group in the unit A, failed to show a cytotoxic effect, suggesting that the isoprenyl group might impede the cytotoxic activity. It’s worth to note that compound **8** showed better cytotoxicity than **7**, which indicated that the position of the olefinic bond should play a vital influence on their biological activity.

## Figures and Tables

**Table 1:** ^1^H (600 MHz) and ^13^C (150 MHz) NMR data of **1** and **2** in CD_3_Cl (*δ* ppm, *J* in Hz).

No.	1		2	
	*δ*_H_ (*J* in Hz)	*δ*_C_	*δ*_H_ (*J* in Hz)	*δ*_C_
1		141.0, C		140.6, C
2		117.4, C		117.3, C
3		160.8, C		160.8, C
4	7.06, d, (8.8)	119.5, CH	7.07, d, (8.7)	119.6, CH
5	7.45, d, (8.8)	138.5, CH	7.45, d, (8.7)	138.7, CH
6		129.9, C		129.9, C
7	3.12, s	31.0, CH_2_	3.12, s	31.0, CH_2_
8	5.05, m	121.9, CH	5.05, m	121.7, CH
9		134.1, C		134.2, C
10	1.47, s	17.8, CH_3_	1.47, s	17.8, CH_3_
11	1.58, s	25.7, CH_3_	1.59, s	25.7, CH_3_
12		203.1, C=O		203.1, C=O
13	9.71, s	194.5, C=O	9.71, s	194.4, C=O
1’		121.0, C		121.3, C
2’		151.7, C		151.6, C
3’		148.0, C		147.7, C
4’	6.93, d, (1.9)	121.8, CH	7.03, s	123.7, CH
5’		128.6, C		129.1, C
6’	6.47, d, (1.9)	123.6, CH	6.56, s	125.1, CH
7’	2.17, s	21.1, CH_3_	2.19, s	21.0, CH_3_
8α’	4.62, d, (5.7)	66.3, CH_2_	4.12, dd, (9.5, 7.8)	71.8, CH_2_
8β’			4.43, dd, (9.5, 2.8)	
9’	5.55, m	119.4, CH	4.07, dd, (7.8, 2.8)	76.6, CH
10’		138.7, C		71.1, C
11’	1.76, s	18.4, CH_3_	1.69, s	29.8, CH_3_
12’	1.80, s	26.0, CH_3_	1.71, s	28.4, CH_3_
3-OH	11.51, s		11.50, s	
2’-OH	12.09, s		12.13, s	

**Table 2:** ^1^H (600 MHz) and ^13^C (150 MHz) NMR data of **3** and **4** (*δ* ppm, *J* in Hz).

No.	3		4	
	*δ*_H_ (*J* in Hz)	*δ*_C_	*δ*_H_ (*J* in Hz)	*δ*_C_
1		140.6, C		141.7, C
2		117.3, C		119.2, C
3		160.8, C		161.1, C
4	7.08, d, (8.7)	119.6, CH	7.11, d, (8.6)	119.6, CH
5	7.46, d, (8.7)	138.7, CH	7.55, d, (8.6)	139.2, CH
6		129.9, C		130.5, C
7	3.13, s	31.0, CH_2_	3.14, d, (7.2)	31.4, CH_2_
8	5.06, m	121.7, CH	5.08, m	122.3, CH
9		134.2, C		133.8, C
10	1.48, s	17.8, CH_3_	1.46, s	17.6, CH_3_
11	1.59, s	25.7, CH_3_	1.55, s	25.7, CH_3_
12		203.2, C=O		203.6, C=O
13	9.72, s	194.4, C=O	9.98, s	194.1, C=O
1’		121.5, C		123.2, C
2’		151.5, C		152.1, C
3’		147.0, C		148.8, C
4’	6.90, d, (1.9)	123.4, CH	7.18, d, (2.0)	123.0, CH
5’		128.8, C		129.2, C
6’	6.57, s	125.4, CH	6.69, s	124.6, CH
7’	2.16, s	21.0, CH_3_	2.16, s	20.7, CH_3_
8α’	4.79, s	72.9, CH_2_	4.01, dd, (9.8, 7.5)	74.3, CH_2_
8β’			4.15, dd, (9.8, 3.9)	
9’		210.6, C=O	4.47, dd, (7.5, 3.9)	74.0, CH
10’	3.00, m	37.2, CH		146.0, C
11α’	1.20, s	18.1, CH_3_	4.92, s	112.2, CH_2_
11β’			5.12, m	
12’	1.21, s	29.9, CH_3_	1.83, s	19.0, CH_3_
3-OH	11.51, s			
2’-OH	12.10, s			

**Table 3:** ^1^H (600 MHz) and ^13^C (150 MHz) NMR data of **5** in CD_3_Cl (*δ* ppm, *J* in Hz).

No.	*δ*_H_ (*J* in Hz)	*δ*_C_	No.	*δ*_H_ (*J* in Hz)	*δ*_C_
1		142.5, C	4’	6.96, s	121.7, CH
2		117.9, C	5’		128.3, C
3		162.9, C	6’	6.69, m	124.2, CH
4	7.16, d, (8.5)	120.5, CH	7’	2.12, s	21.2, CH_3_
5	7.59, dd, (8.5, 7.3)	136.1, CH	8’	4.62, d, (7.0)	66.3, CH_2_
6	6.98, d, (7.3)	120.0, CH	9’	5.55, m	119.5, CH
7		201.0, C=O	10’		138.8, C
8	9.91, s	195.0, C=O	11	1.76, s	18.4, CH_3_
1’		119.9, C	12’	1.80, s	26.0, CH_3_
2’		152.3, C	3-OH	11.74, s	
3’		148.1, C	2’-OH	11.93, s	

**Table 4:** ^1^H (500 MHz) and ^13^C (125 MHz) NMR data of **7** in CD_3_OD (*δ* ppm, *J* in Hz).

No.	*δ*_H_ (*J* in Hz)	*δ*_C_	No.	*δ*_H_ (*J* in Hz)	*δ*_C_
1α	2.34, m	30.2, CH_2_	13	1.85, s	22.3, CH_3_
1β	2.45, m		14	0.98, d, (6.7)	10.8, CH_3_
2α	1.46, m	31.7, CH_2_	15	1.03, s	17.3, CH_3_
2β	2.16, overlapped		1’		175.2, C=O
3	4.87, td, (11.2, 4.4)	74.2, CH	2’	2.56, m	46.0, CH
4	1.67, dt, (11.2, 6.7)	46.1, CH	3’	4.24, m	74.5, CH
5		42.3, C	4’	5.59, dd, (15.0, 7.0)	131.5, CH
6α	2.16, overlapped	41.2, CH_2_	5’	6.27, dd, (15.0, 10.4)	132.5, CH
6β	2.91, d, (13.7)		6’	6.12, dd, (15.0, 10.4)	132.5, CH
7		127.2, C	7’	5.72, dd, (15.0, 7.0)	131.5, CH
8		191.7, C=O	8’	2.26, m	42.7, CH_2_
9	5.77, d, (1.8)	126.9, CH	9’	3.86, m	67.5, CH
10		164.9, C	10’	1.20, d (6.2)	23.1, CH_3_
11		143.7, C	11’	1.18, d (7.2)	14.3, CH_3_
12	2.10, s	22.8, CH_3_			

**Table 5:** Cytotoxic activities of compounds **1**–**8**.

Compounds	IC_50_ (μM) ^a^			
	HepG-2	MCF-7	SF-268	A549
**1**	>100	>100	>100	>100
**2**	>100	>100	>100	>100
**3**	>100	>100	>100	>100
**4**	88.6 ± 3.1	85.7 ± 7.4	67.7 ± 3.1	>100
**5**	16.0 ± 0.1	25.1 ± 1.1	23.0 ± 0.9	17.6 ± 0.3
**6**	>100	>100	>100	>100
**7**	90.9 ± 2.0	81.1 ± 2.8	92.5 ± 4.3	59.2 ± 2.1
**8**	26.2 ± 0.8	29.6 ± 4.6	28.8 ± 0.2	25.5 ± 0.4
cisplatin	2.4 ± 0.1	3.2 ± 0.1	3.3 ± 0.3	1.6 ± 0.1

^a^ Values are expressed as the mean ± SD.

## Supplementary Materials

The following are available online at http://www.mdpi.com/1660-3397/16/9/329/s1. Figures S1–S4: HRESIMS, IR, UV, CD and 1D and 2D NMR spectra of compounds **1**–**5**, and **7**; Figures S55–S58: 1D NMR spectra of compounds **6** and **8**.
