# Supplementary material for: Highly Substituted Benzophenone Aldehydes and Eremophilane Derivatives from the Deep-Sea Derived Fungus Phomopsis lithocarpus FS508
Source: Mar Drugs. 2018 Sep 11;16(9):329. doi: 10.3390/md16090329 (PMC6165036; doi:10.3390/md16090329)
Supplement: Supplementary file 1 [file marinedrugs-16-00329-s001.pdf]

# Supplementary Materials

## Highly substituted Benzophenone Aldehydes and eremophilane derivatives from the Deep-sea Derived Fungus *Phomopsis lithocarpus* FS508

Jian-Lin Xu [1,2,†], Hong-Xin Liu [1,3,†], Yu-Chan Chen [1], Hai-Bo Tan [3], Heng Guo [1,2], Li-Qiong Xu [1], Sai-Ni Li [1], Zi-Lei Huang [1], Hao-Hua Li [1], Xiao-Xia Gao [2,*] and Wei-Min Zhang [1,*]

[1]State Key Laboratory of Applied Microbiology Southern China, Guangdong Provincial Key Laboratory of Microbial Culture Collection and Application, Guangdong Open Laboratory of Applied Microbiology, Guangdong Institute of Microbiology, Guangzhou 510070, China

[2]College of Pharmacy, Guangdong Pharmaceutical University, Guangzhou 510006, China

[3]Program for Natural Products Chemical Biology, Key Laboratory of Plant Resources Conservation and Sustainable Utilization, Guangdong Provincial Key Laboratory of Applied Botany, South China Botanical Garden, Chinese Academy of Sciences, Guangzhou 510650, China

# Table of Contents


Figure S1. HRESIMS spectrum of tenellone D (**1**)....4
Figure S2. $^{1}H$ NMR spectrum (600 MHz, $CD_3Cl$) of tenellone D (**1**). ....4
Figure S3. $^{13}C$ NMR spectrum (150 MHz, $CD_3Cl$) of tenellone D (**1**). ....5
Figure S4. $^{1}H$-$^{1}H$ COSY spectrum (600 MHz, $CD_3Cl$) of tenellone D (**1**). ....5
Figure S5. HSQC spectrum of tenellone D (**1**). ....6
Figure S6. HMBC spectrum of tenellone D (**1**). ....6
Figure S7. UV spectrum of tenellone D (**1**) ....7
Figure S8. IR spectrum of tenellone D (**1**)....7
Figure S9. HRESIMS spectrum of tenellone E (**2**). ....8
Figure S10. $^{1}H$ NMR spectrum (600 MHz, $CD_3Cl$) of tenellone E (**2**). ....8
Figure S11. $^{13}C$ NMR spectrum (150 MHz, $CD_3Cl$) of tenellone E (**2**). ....9
Figure S12. $^{1}H$-$^{1}H$ COSY spectrum (600 MHz, $CD_3Cl$) of tenellone E (**2**). ....9
Figure S13. HSQC spectrum of tenellone E (**2**). ....10
Figure S14. HMBC spectrum of tenellone E (**2**). ....10
Figure S15. NOESY spectrum (600 MHz, $CD_3Cl$) of tenellone E (**2**). ....11
Figure S16. CD spectrum of tenellone E (**2**). ....11
Figure S17. UV spectrum of tenellone E (**2**)....12
Figure S18. IR spectrum of tenellone E (**2**) ....12
Figure S19. HRESIMS spectrum of tenellone F (**3**). ....13
Figure S20. $^{1}H$ NMR spectrum (600 MHz, $CD_3Cl$) of tenellone F (**3**). ....13
Figure S21. $^{13}C$ NMR spectrum (150 MHz, $CD_3Cl$) of tenellone F (**3**). ....14
Figure S22. $^{1}H$-$^{1}H$ COSY spectrum (600 MHz, $CD_3Cl$) of tenellone F (**3**). ....14
Figure S23. HSQC spectrum of tenellone F (**3**). ....15
Figure S24. HMBC spectrum of tenellone F (**3**). ....15
Figure S25. UV spectrum of tenellone F (**3**) ....16
Figure S26. IR spectrum of tenellone F (**3**)....16
Figure S27. HRESIMS spectrum of tenellone G (**4**)....17
Figure S28. $^{1}H$ NMR spectrum (600 MHz, $CD_3COCD_3$) of tenellone G (**4**)....17
Figure S29. $^{13}C$ NMR spectrum (150 MHz, $CD_3COCD_3$) of tenellone G (**4**)....18
Figure S30. $^{1}H$-$^{1}H$ COSY spectrum (600 MHz, $CD_3COCD_3$) of tenellone G (**4**)....18
Figure S31. HSQC spectrum of tenellone G (**4**). ....19
Figure S32. HMBC spectrum of tenellone G (**4**). ....19
Figure S33. NOESY spectrum (600 MHz, $CD_3COCD_3$) of tenellone G (**4**)....20
Figure S34. CD spectrum of tenellone G (**4**). ....20

Figure S35. UV spectrum of tenellone G (**4**). ....................21
Figure S36. IR spectrum of tenellone G (**4**). ....................21
Figure S37. HRESIMS spectrum of tenellone H (**5**). ....................22
Figure S38. $^{1}$H NMR spectrum (600 MHz, $CD_3Cl$) of tenellone H (**5**). ....................22
Figure S39. $^{13}$C NMR spectrum (150 MHz, $CD_3Cl$) of tenellone H (**5**). ....................23
Figure S40. $^{1}$H-$^{1}$H COSY spectrum (600 MHz, $CD_3Cl$) of tenellone H (**5**). ....................23
Figure S41. HSQC spectrum of tenellone H (**5**). ....................24
Figure S42. HMBC spectrum of tenellone H (**5**). ....................24
Figure S43. UV spectrum of tenellone H (**5**). ....................25
Figure S44. IR spectrum of tenellone H (**5**). ....................25
Figure S45. HRESIMS spectrum of lithocarin A (**7**). ....................26
Figure S46. $^{1}$H NMR spectrum (500 MHz, $CD_3OD$) of lithocarin A (**7**). ....................26
Figure S47. $^{13}$C NMR spectrum (125 MHz, $CD_3OD$) of lithocarin A (**7**). ....................27
Figure S48. $^{1}$H-$^{1}$H COSY spectrum (500 MHz, $CD_3OD$) of lithocarin A (**7**). ....................27
Figure S49. HSQC spectrum of lithocarin A (**7**). ....................28
Figure S50. HMBC spectrum of lithocarin A (**7**). ....................28
Figure S51. NOESY spectrum (500 MHz, $CD_3OD$) of lithocarin A (**7**). ....................29
Figure S52. CD spectrum of lithocarin A (**7**). ....................29
Figure S53. UV spectrum of lithocarin A (**7**). ....................30
Figure S54. IR spectrum of lithocarin A (**7**). ....................30
Figure S55. $^{1}$H NMR spectrum (500 MHz, $CD_3OD$) of tenellone A (**6**). ....................31
Figure S56. $^{13}$C NMR spectrum (125 MHz, $CD_3OD$) of tenellone A (**6**). ....................31
Figure S57. $^{1}$H NMR spectrum (500 MHz, $CD_3OD$) of AA03390 (**8**). ....................32
Figure S58. $^{13}$C NMR spectrum (125 MHz, $CD_3OD$) of AA03390 (**8**). ....................32

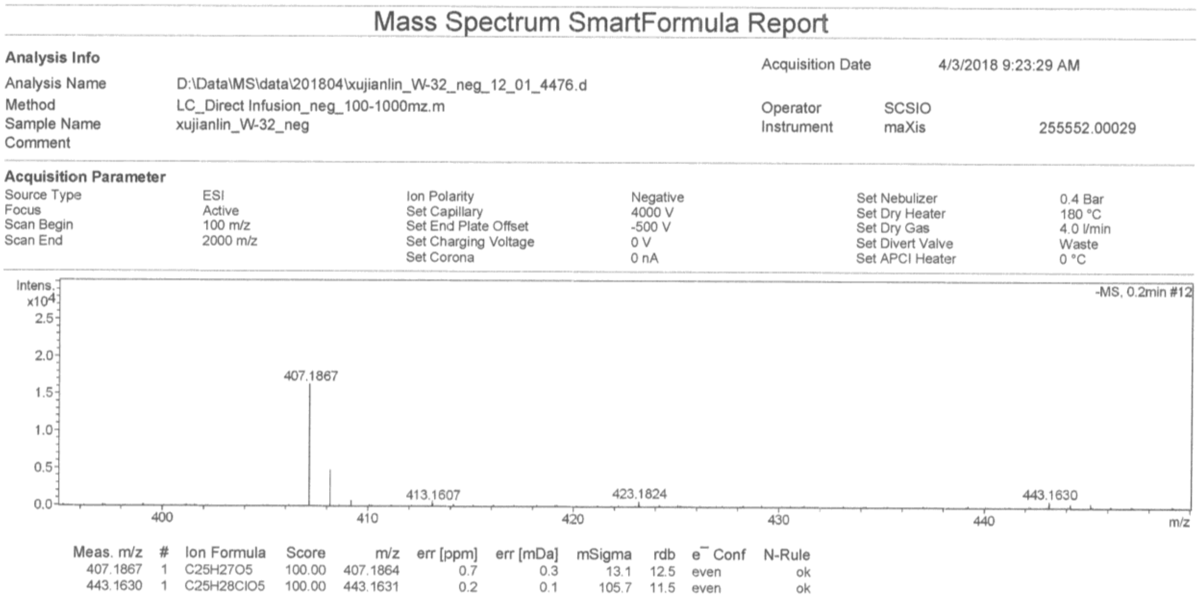


Figure S1. HRESIMS spectrum of tenellone D (**1**).

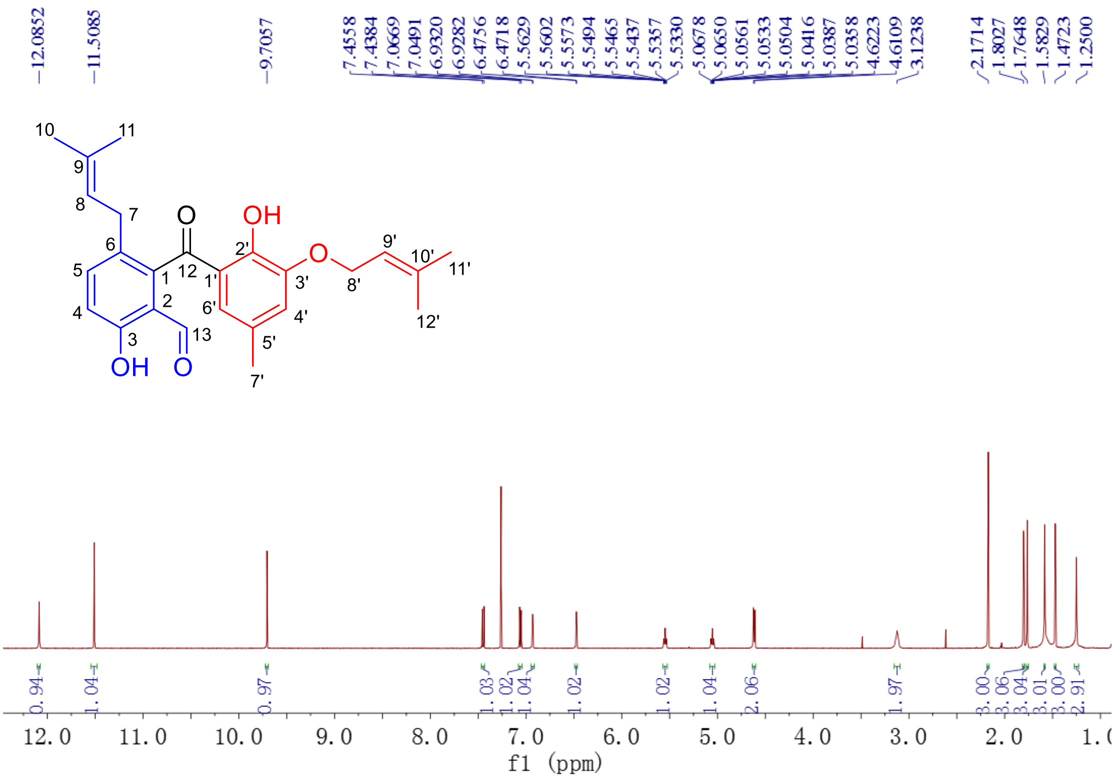


Figure S2. $^{1}$H NMR spectrum (600 MHz, CD$_3$Cl) of tenellone D (**1**).

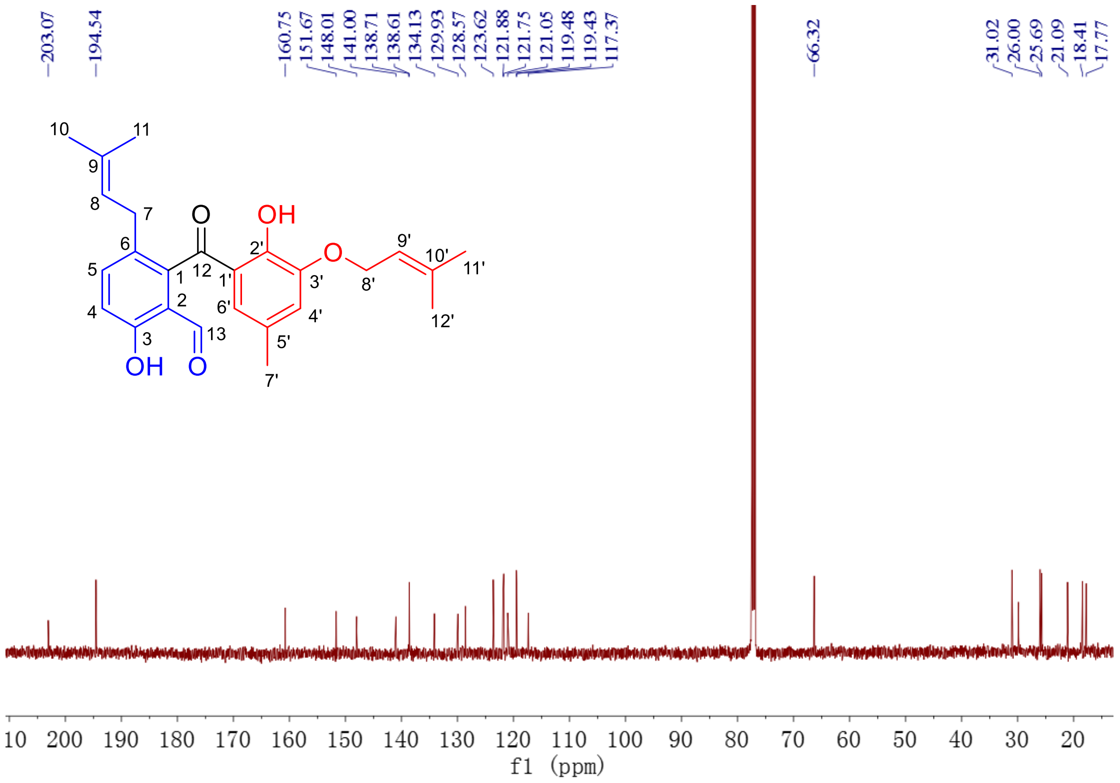


Figure S3. $^{13}$C NMR spectrum (150 MHz, CD3Cl) of tenellone D (**1**).

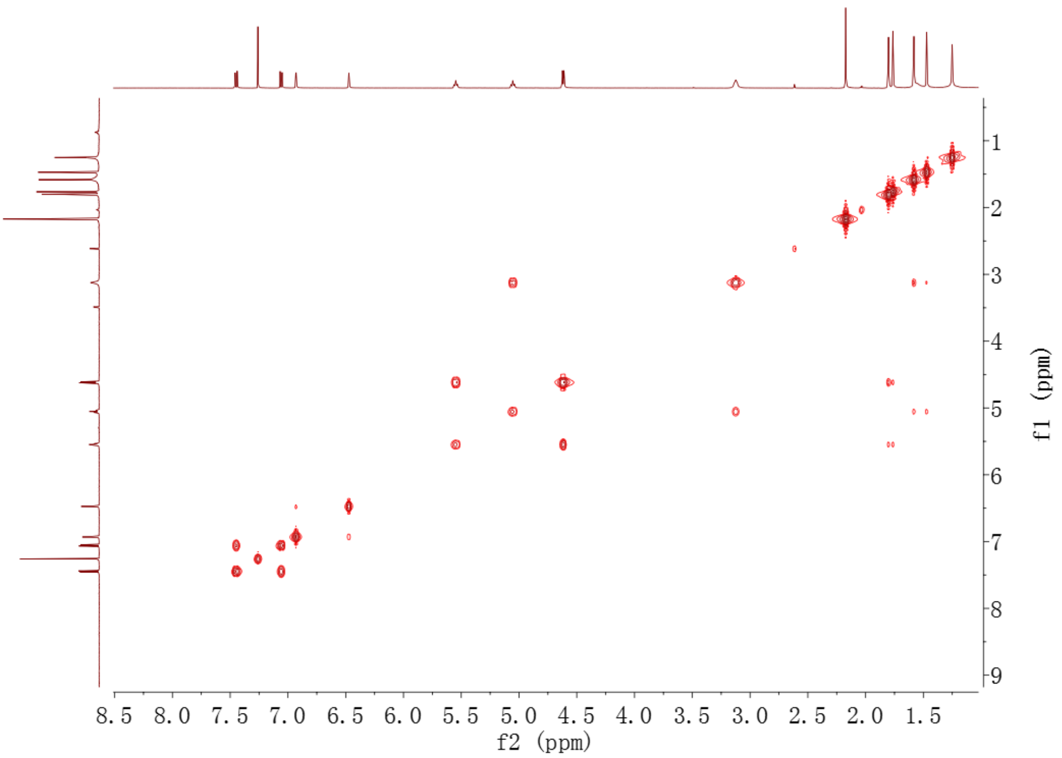


Figure S4. $^{1}$H-$^{1}$H COSY spectrum (600 MHz, CD3Cl) of tenellone D (**1**).

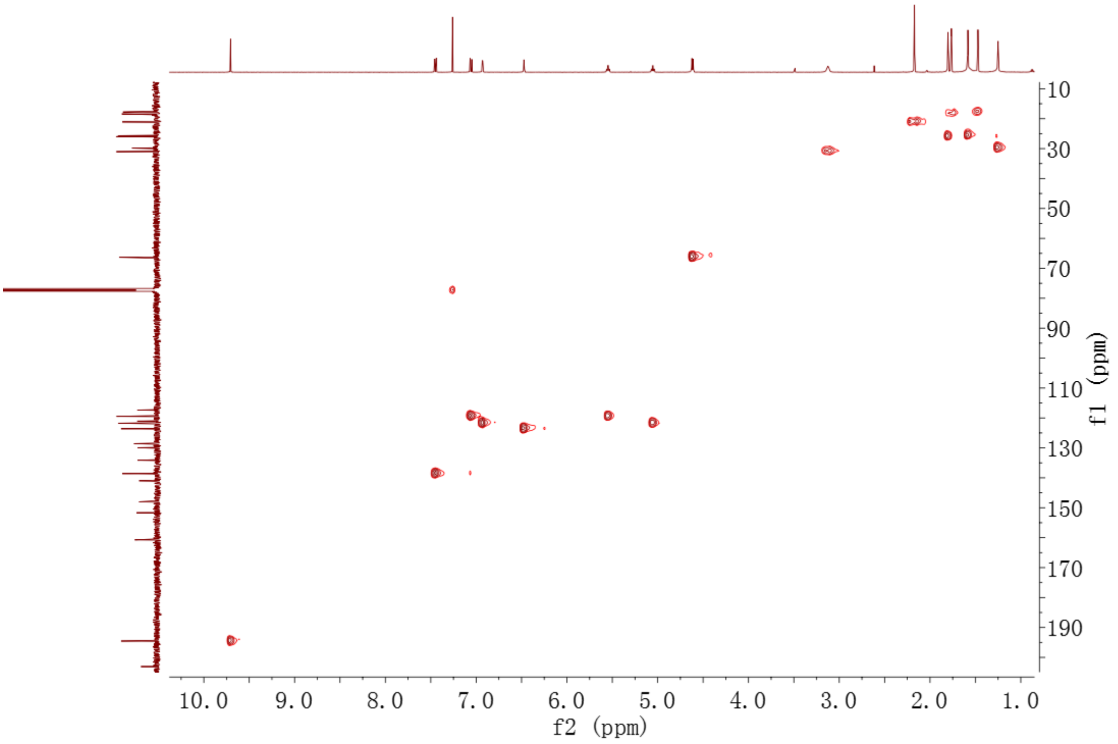


Figure S5. HSQC spectrum of tenellone D (**1**).

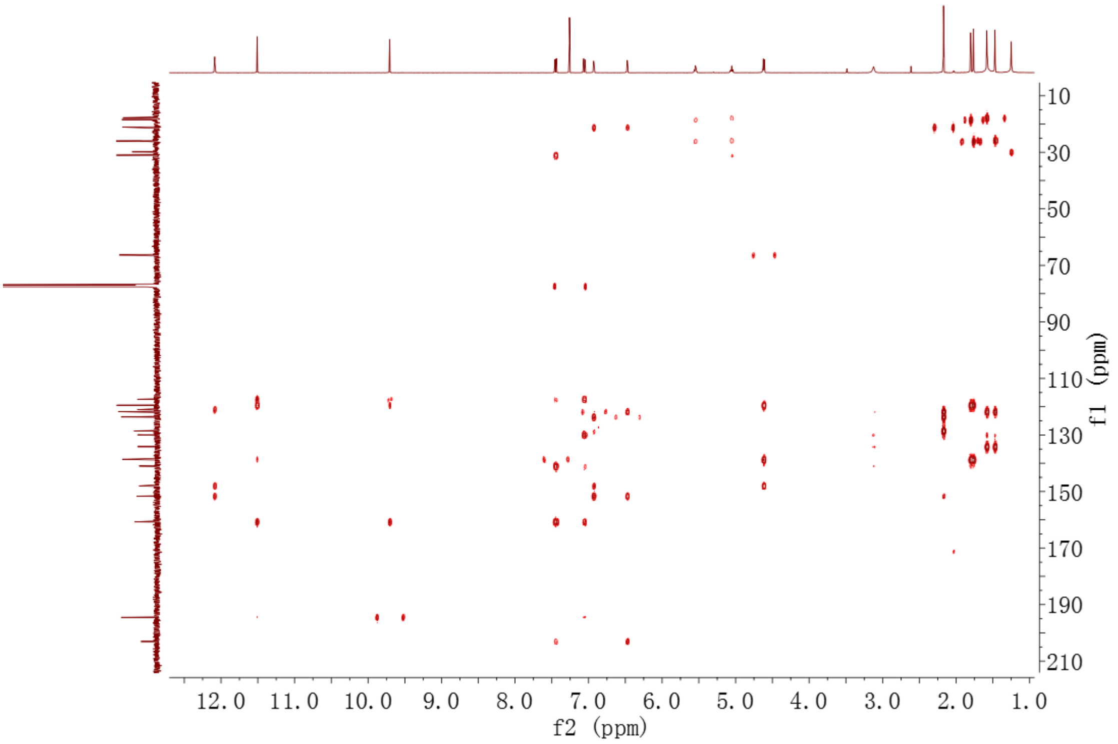


Figure S6. HMBC spectrum of tenellone D (**1**).

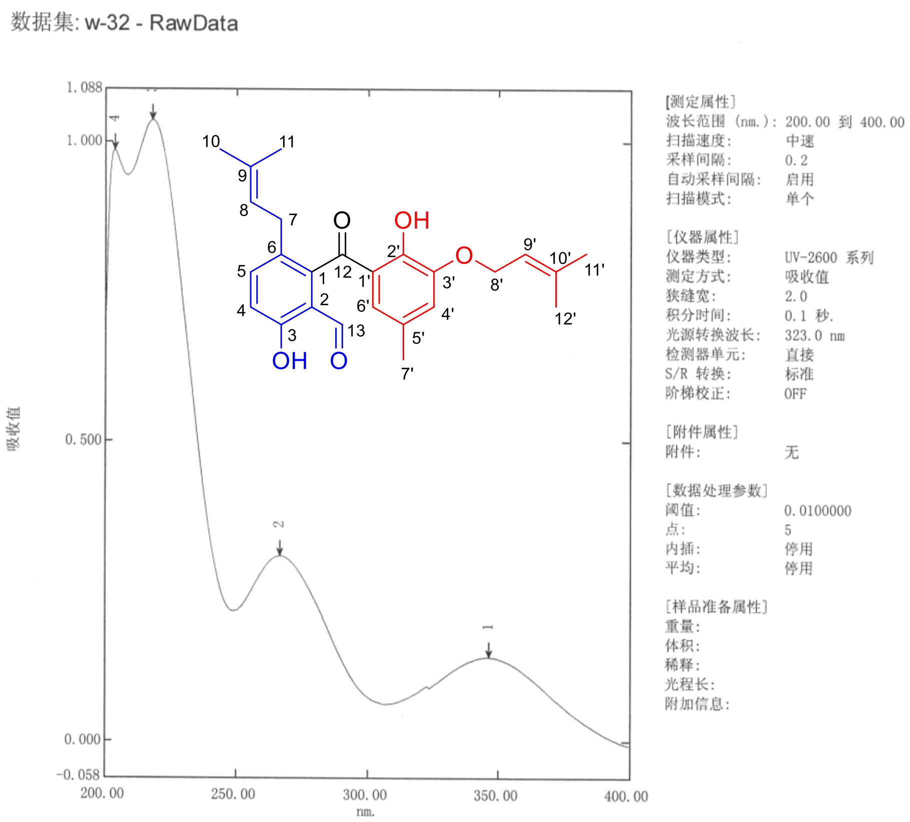


Figure S7. UV spectrum of tenellone D (**1**).

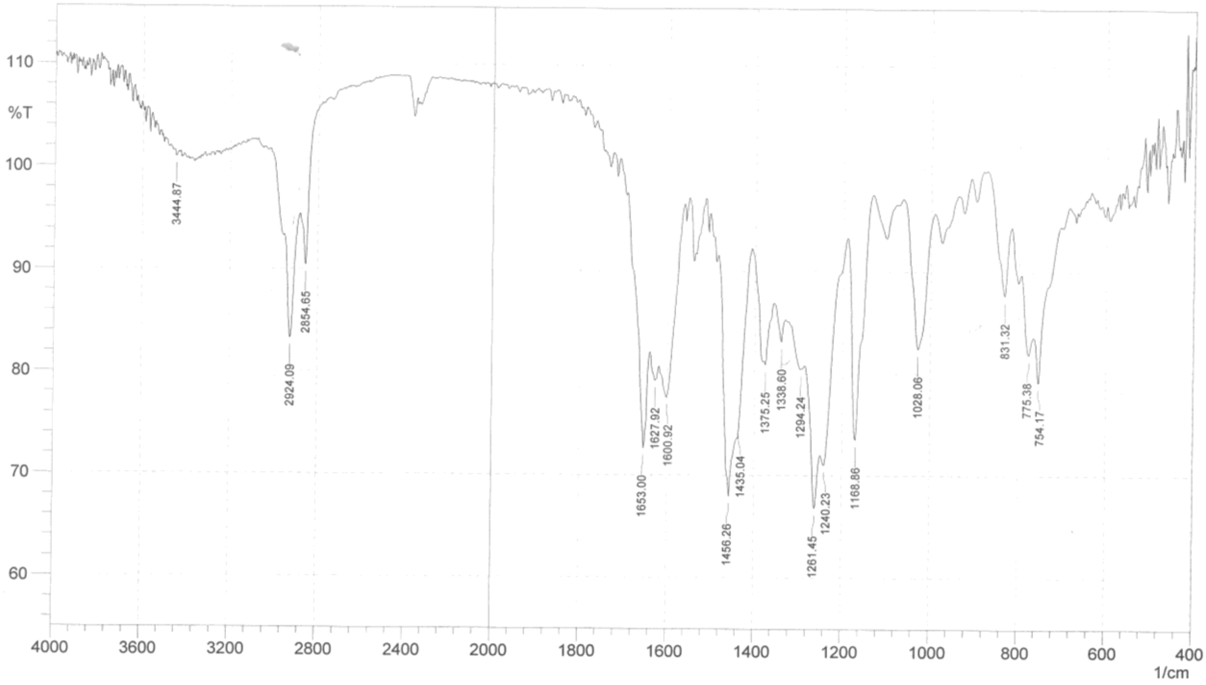


Figure S8. IR spectrum of tenellone D (**1**).

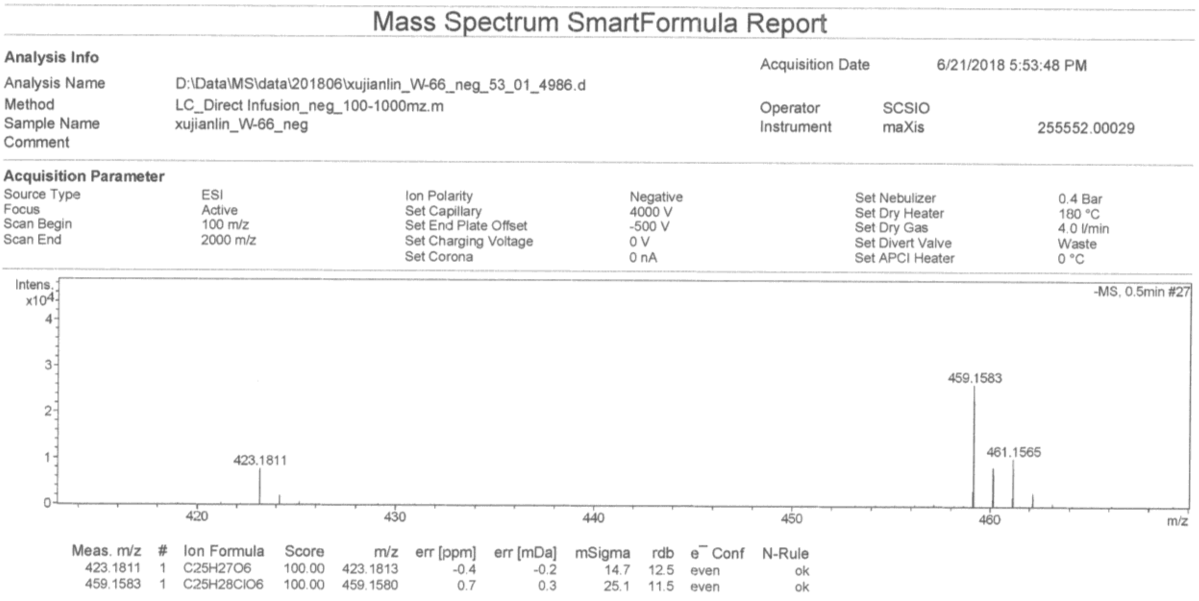


Figure S9. HRESIMS spectrum of tenellone E (**2**).

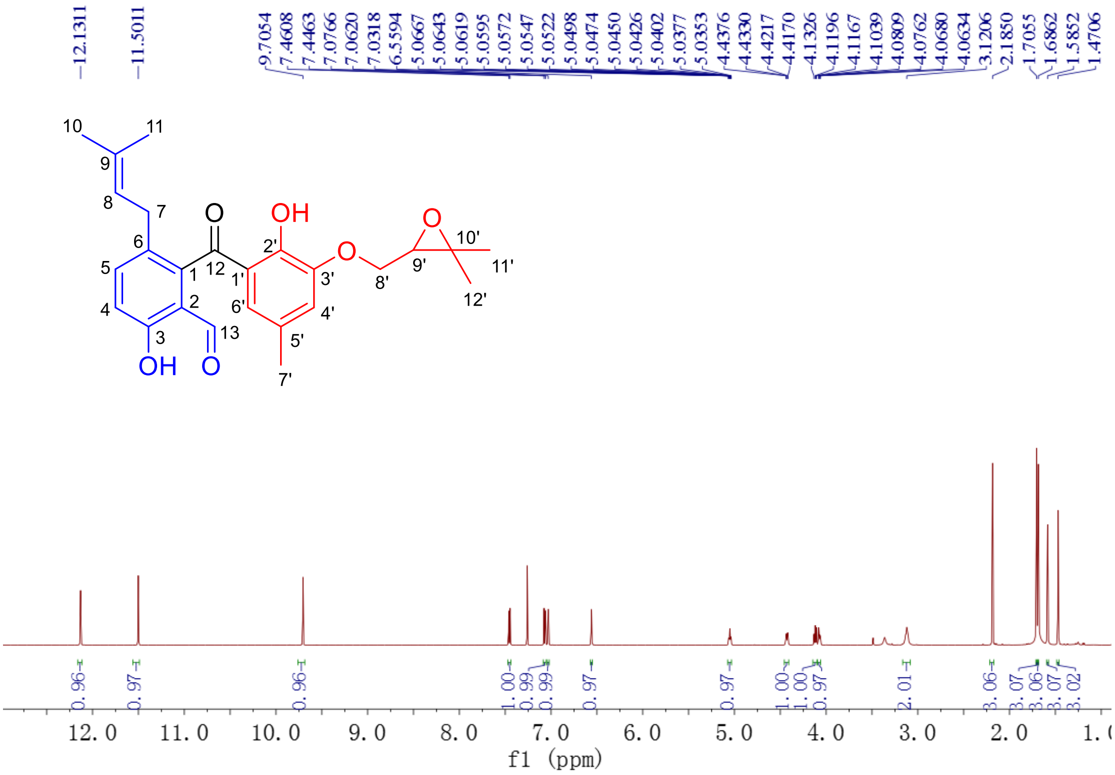


Figure S10. $^{1}$H NMR spectrum (600 MHz, $CD_3Cl$) of tenellone E (**2**).

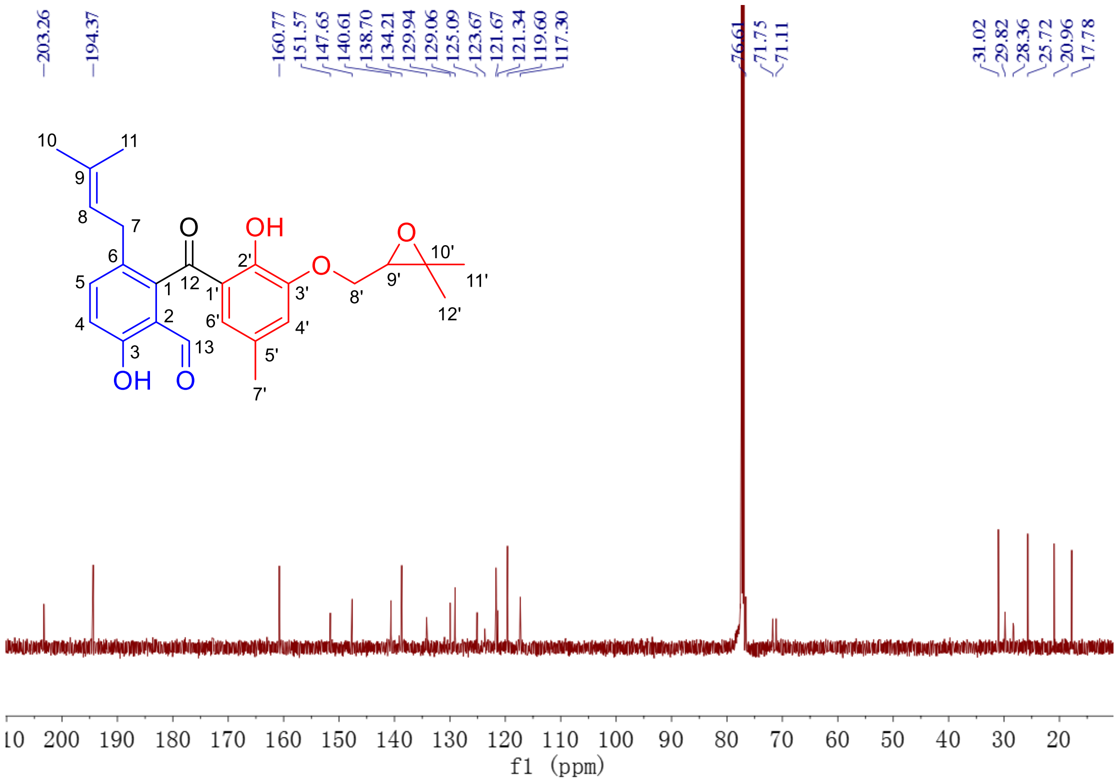


Figure S11. $^{13}$C NMR spectrum (150 MHz, $CD_3Cl$) of tenellone E (**2**).

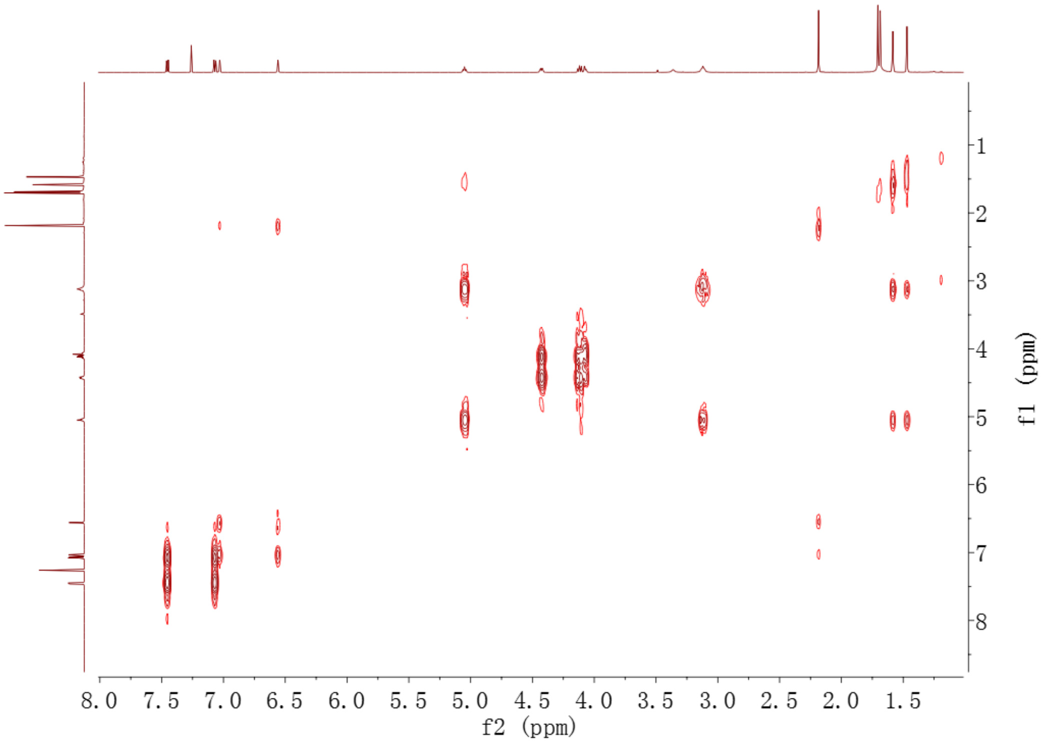


Figure S12. $^{1}$H-$^{1}$H COSY spectrum (600 MHz, $CD_3Cl$) of tenellone E (**2**).

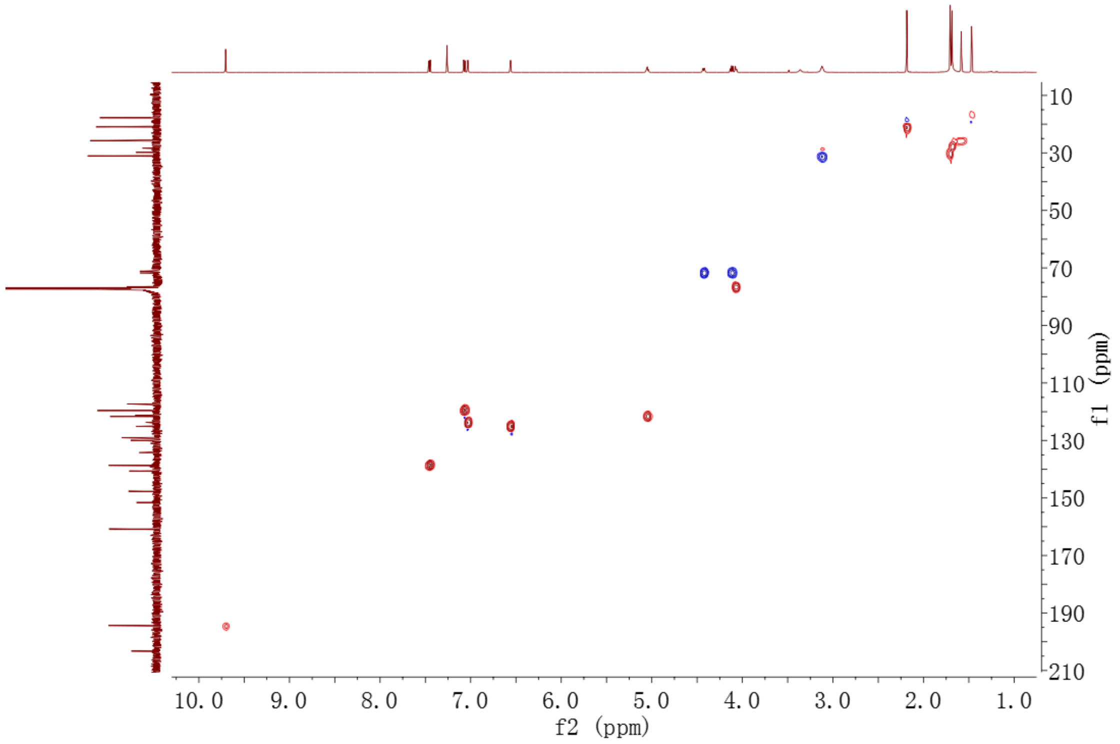


Figure S13. HSQC spectrum of tenellone E (**2**).

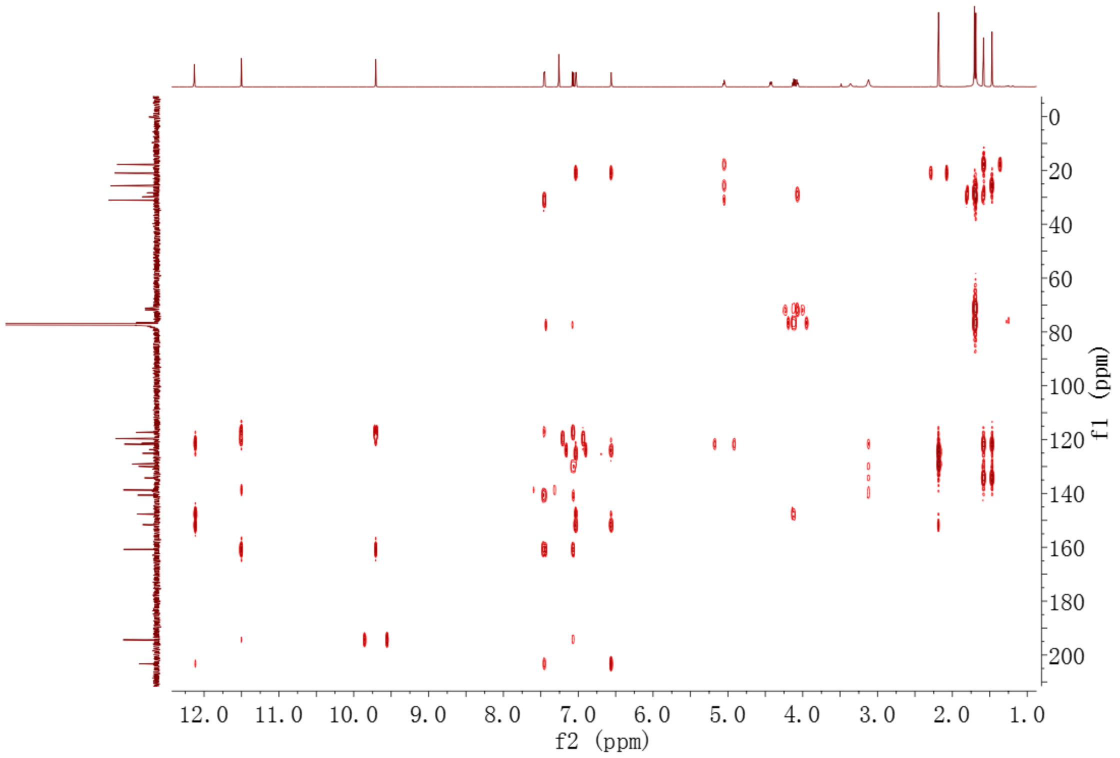


Figure S14. HMBC spectrum of tenellone E (**2**).

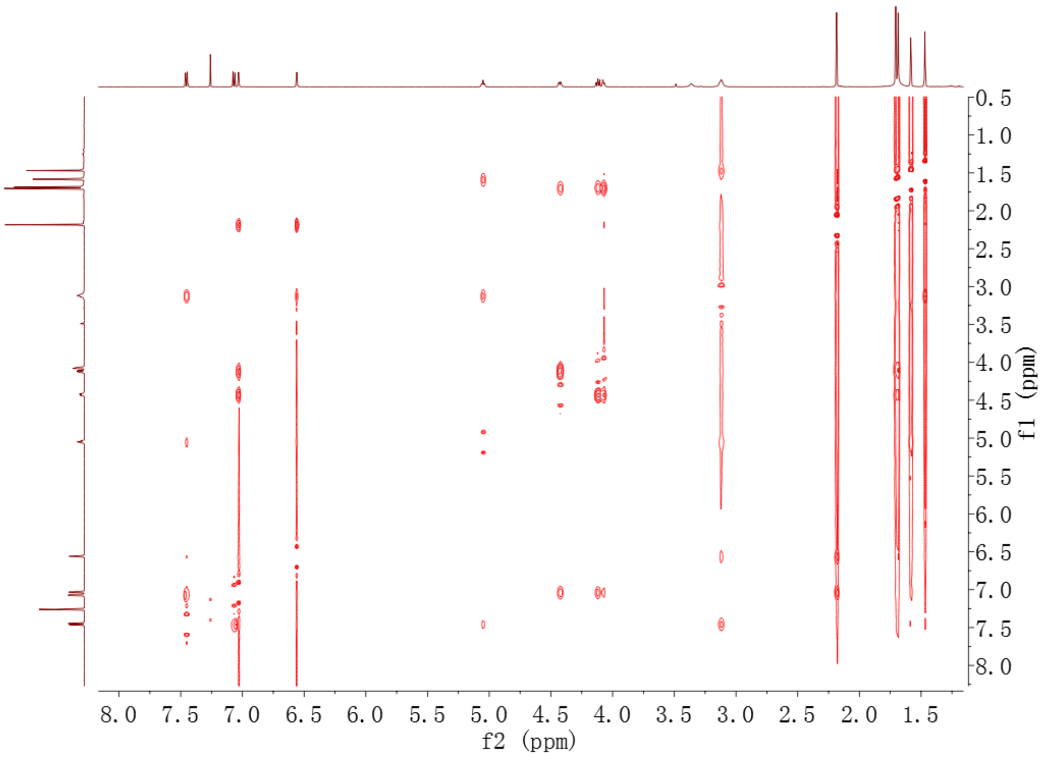


Figure S15. NOESY spectrum (600 MHz, $CD_3Cl$) of tenellone E (**2**).

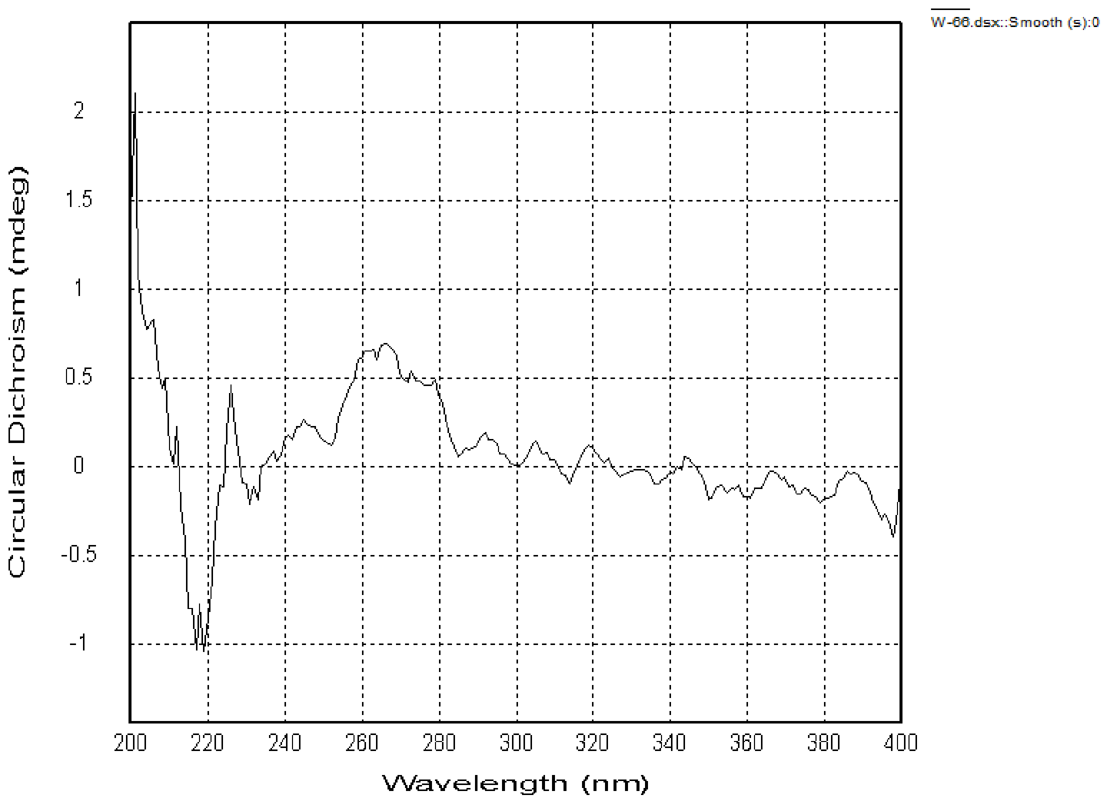


Figure S16. CD spectrum of tenellone E (**2**).

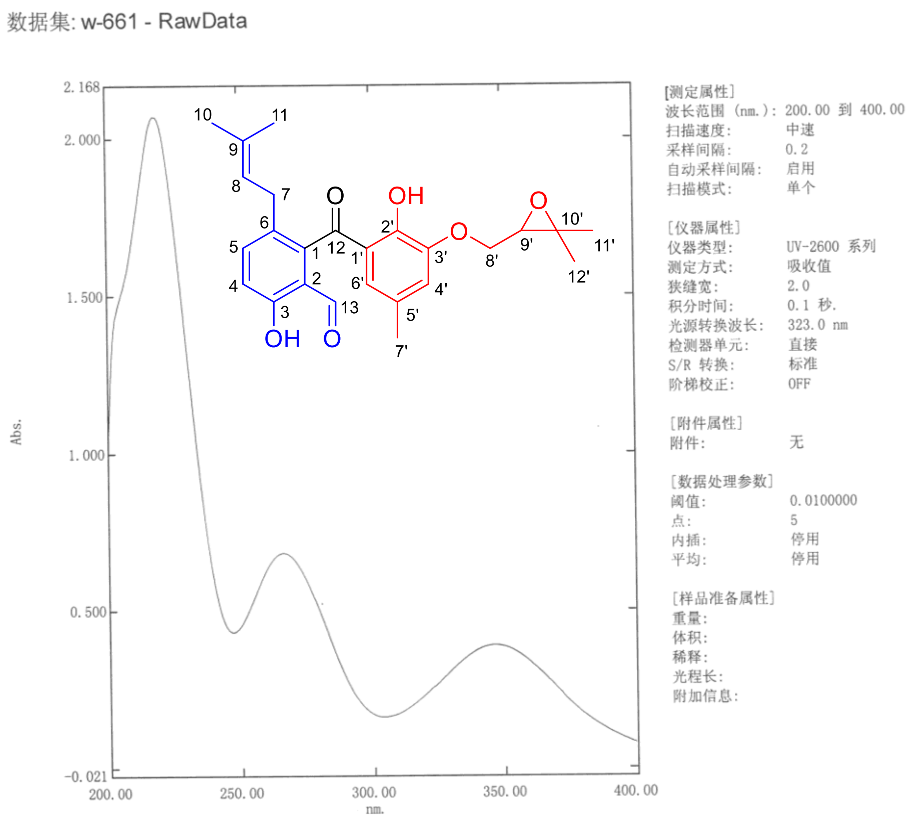


Figure S17. UV spectrum of tenellone E (**2**).

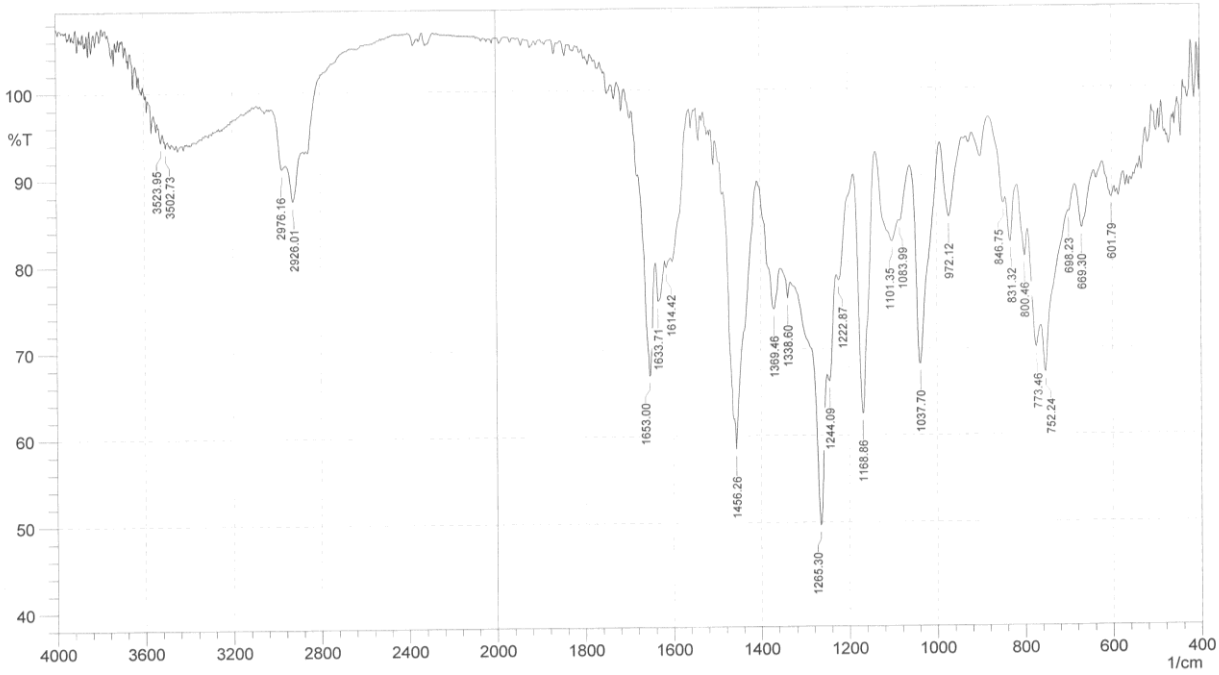


Figure S18. IR spectrum of tenellone E (**2**).

## Mass Spectrum SmartFormula Report

**Analysis Info**

| | | | |
|---|---|---|---|
| Analysis Name | D:\Data\MS\data\201806\xujianlin_W-65_neg_54_01_4987.d | Acquisition Date | 6/21/2018 5:57:19 PM |
| Method | LC_Direct Infusion_neg_100-1000mz.m | Operator | SCSIO |
| Sample Name | xujianlin_W-65_neg | Instrument | maXis 255552.00029 |
| Comment | | | |

**Acquisition Parameter**

| | | | | | |
|---|---|---|---|---|---|
| Source Type | ESI | Ion Polarity | Negative | Set Nebulizer | 0.4 Bar |
| Focus | Active | Set Capillary | 4000 V | Set Dry Heater | 180 °C |
| Scan Begin | 100 m/z | Set End Plate Offset | -500 V | Set Dry Gas | 4.0 l/min |
| Scan End | 2000 m/z | Set Charging Voltage | 0 V | Set Divert Valve | Waste |
| | | Set Corona | 0 nA | Set APCI Heater | 0 °C |

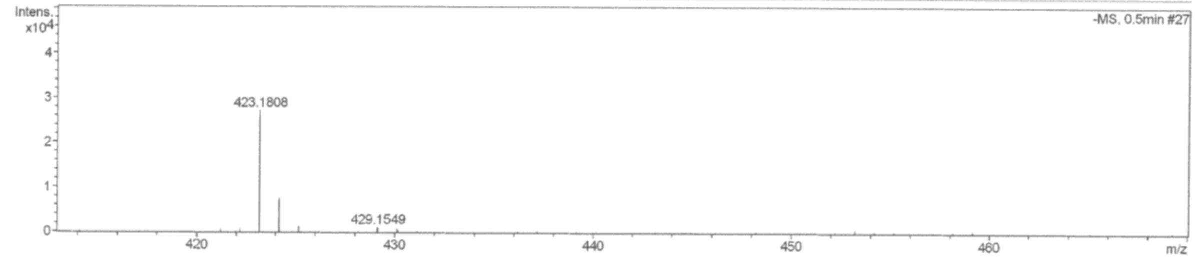


| Meas. m/z | # | Ion Formula | Score | m/z | err [ppm] | err [mDa] | mSigma | rdb | e⁻ Conf | N-Rule |
|---|---|---|---|---|---|---|---|---|---|---|
| 423.1808 | 1 | C25H27O6 | 100.00 | 423.1813 | 1.2 | 0.5 | 3.2 | 12.5 | even | ok |
| 459.1586 | 1 | C25H28ClO6 | 100.00 | 459.1580 | -1.4 | -0.6 | 24.3 | 11.5 | even | ok |
| 847.3675 | 1 | C50H55O12 | 100.00 | 847.3699 | -2.8 | -2.4 | 42.3 | 23.5 | even | ok |

Figure S19. HRESIMS spectrum of tenellone F (**3**).

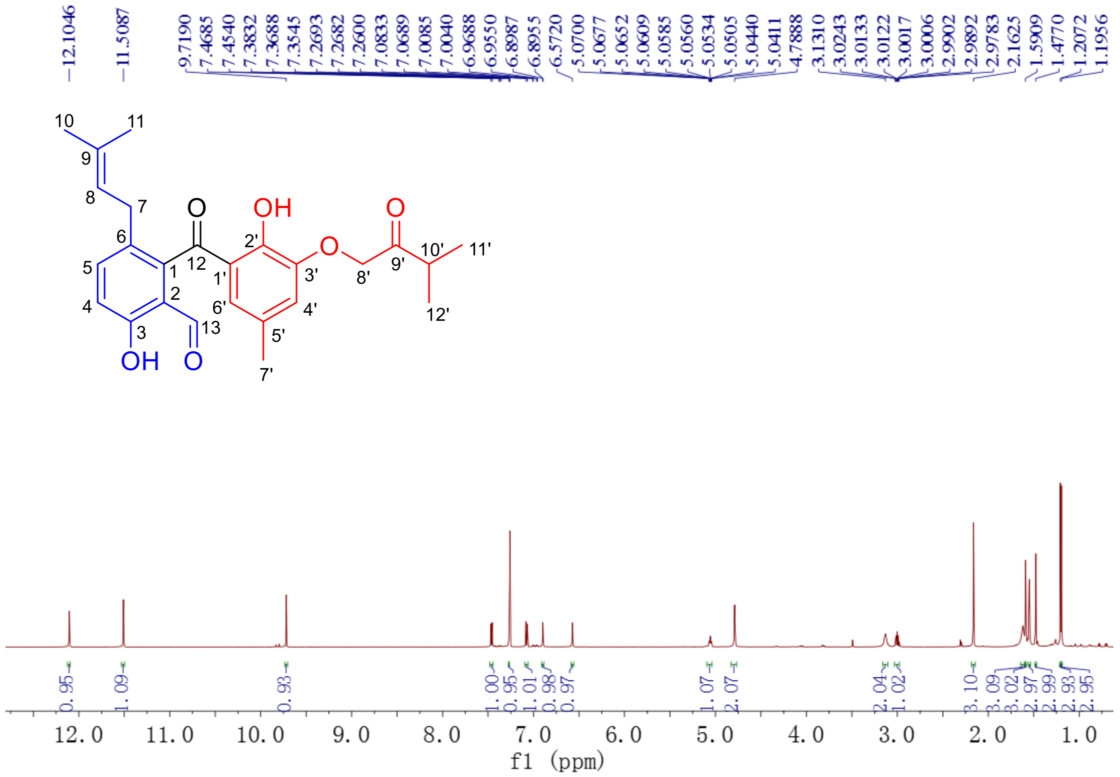


Figure S20. $^{1}$H NMR spectrum (600 MHz, $CD_3Cl$) of tenellone F (**3**).

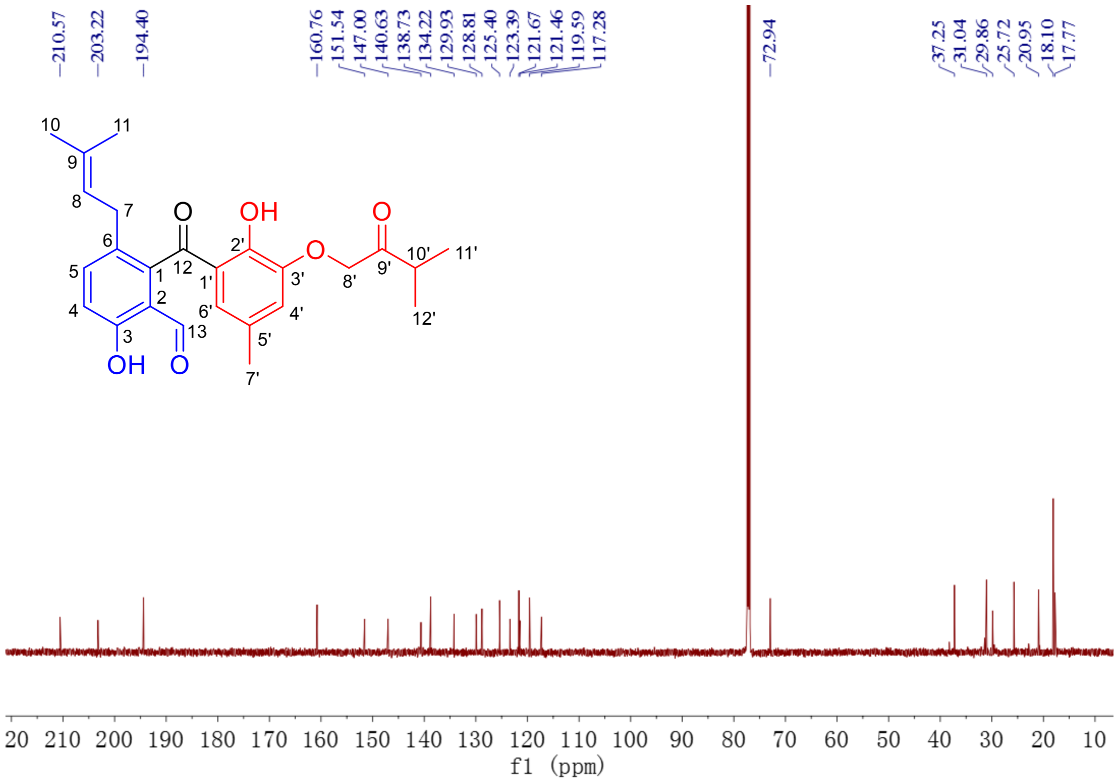

Figure S21. $^{13}C$ NMR spectrum (150 MHz, $CD_3Cl$) of tenellone F (**3**).

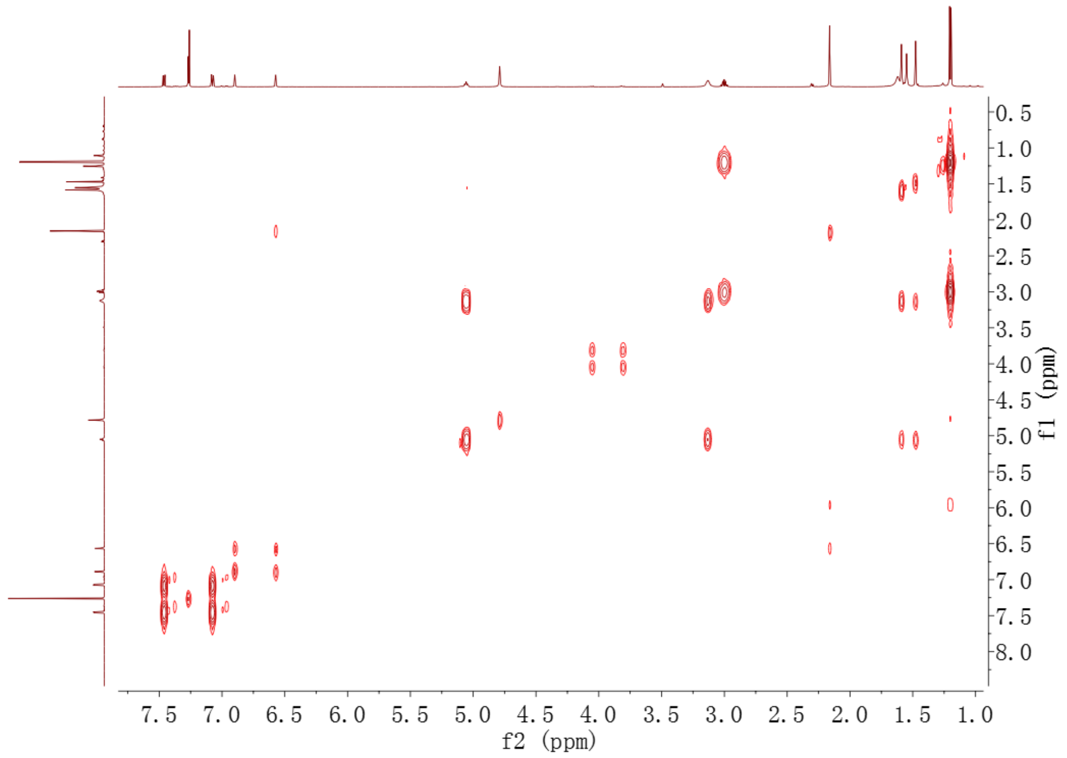

Figure S22. $^1H$-$^1H$ COSY spectrum (600 MHz, $CD_3Cl$) of tenellone F (**3**).

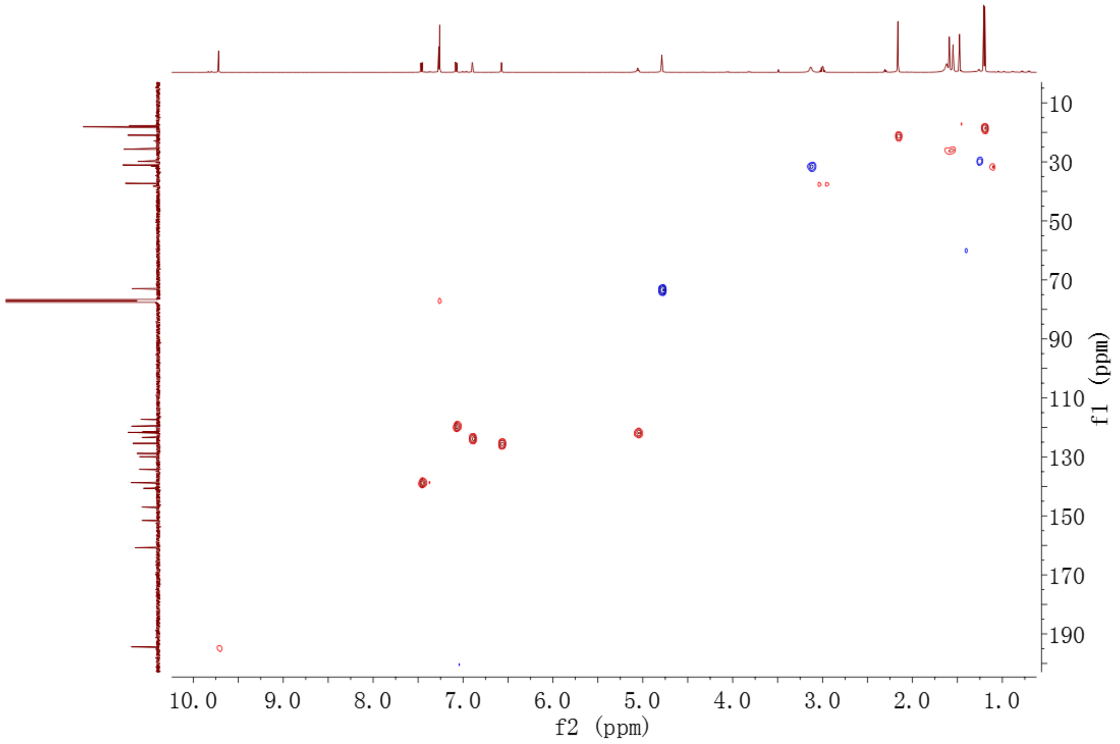


Figure S23. HSQC spectrum of tenellone F (**3**).

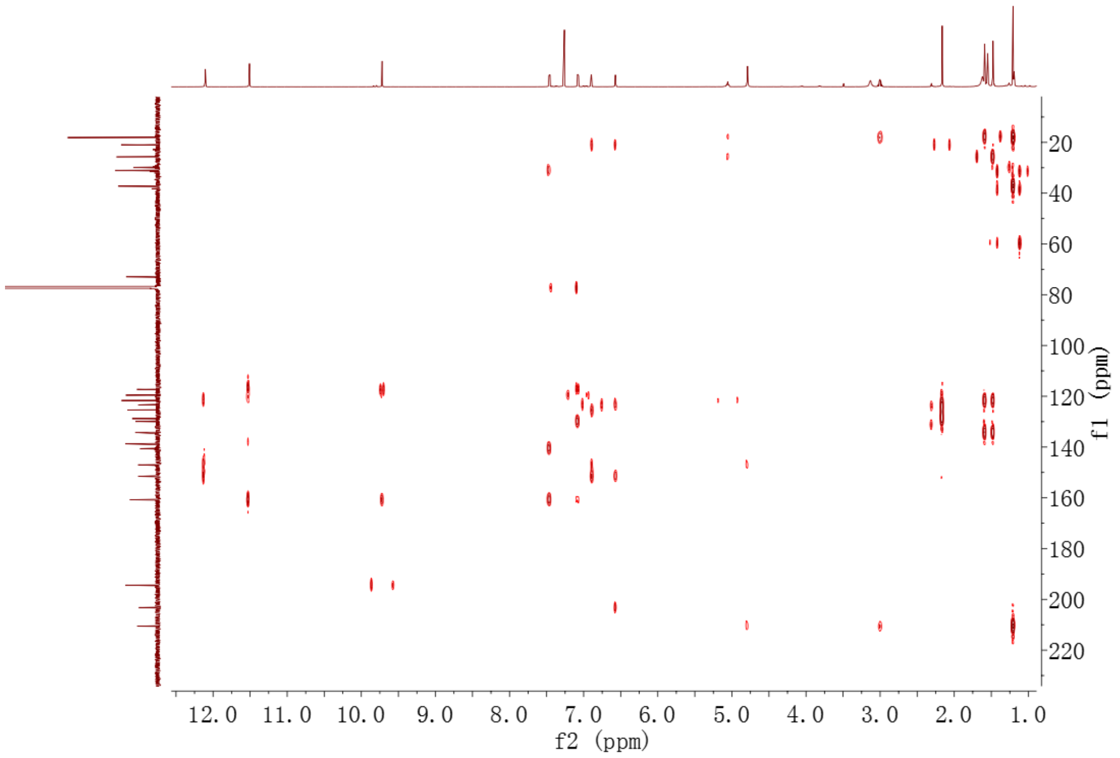


Figure S24. HMBC spectrum of tenellone F (**3**).

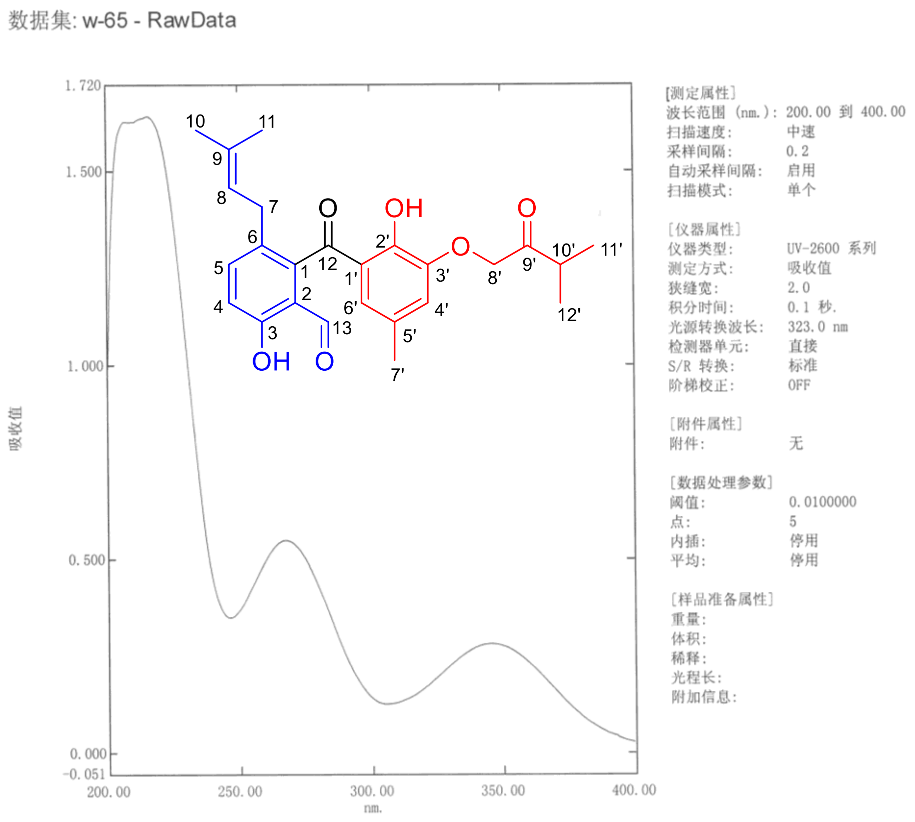


Figure S25. UV spectrum of tenellone F (**3**).

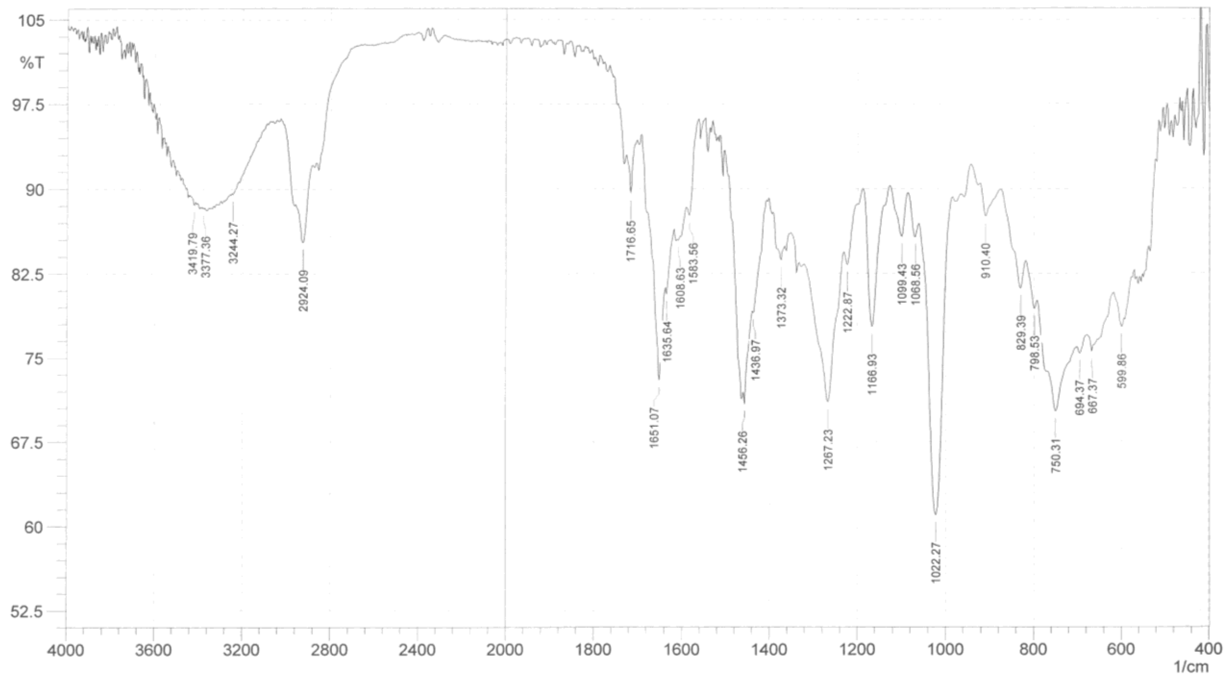


Figure S26. IR spectrum of tenellone F (**3**).

## Mass Spectrum SmartFormula Report

**Analysis Info**

| | | | |
|---|---|---|---|
| Analysis Name | D:\Data\MS\data\201805\xujianlin_W-58_neg_2_01_4698.d | Acquisition Date | 5/8/2018 8:59:40 AM |
| Method | LC_Direct Infusion_neg_100-1000mz.m | Operator | SCSIO |
| Sample Name | xujianlin_W-58_neg | Instrument | maXis 255552.00029 |
| Comment | | | |

**Acquisition Parameter**

| | | | | | |
|---|---|---|---|---|---|
| Source Type | ESI | Ion Polarity | Negative | Set Nebulizer | 0.4 Bar |
| Focus | Active | Set Capillary | 4000 V | Set Dry Heater | 180 °C |
| Scan Begin | 100 m/z | Set End Plate Offset | -500 V | Set Dry Gas | 4.0 l/min |
| Scan End | 2000 m/z | Set Charging Voltage | 0 V | Set Divert Valve | Waste |
| | | Set Corona | 0 nA | Set APCI Heater | 0 °C |

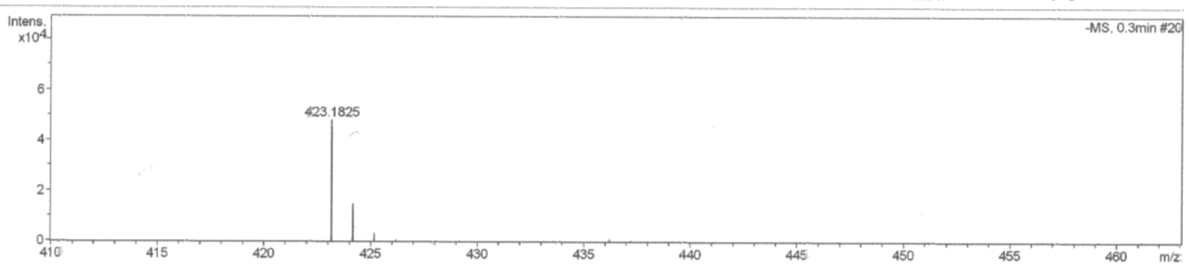


| Meas. m/z | # | Ion Formula | Score | m/z | err [ppm] | err [mDa] | mSigma | rdb | e⁻ Conf | N-Rule |
|---|---|---|---|---|---|---|---|---|---|---|
| 423.1825 | 1 | C25H27O6 | 100.00 | 423.1813 | 2.8 | 1.2 | 19.5 | 12.5 | even | ok |
| 459.1575 | 1 | C25H28ClO6 | 100.00 | 459.1580 | -1.1 | -0.5 | 36.6 | 11.5 | even | ok |
| 847.3698 | 1 | C50H55O12 | 100.00 | 847.3699 | 0.1 | 0.1 | 503.2 | 23.5 | even | ok |

Figure S27. HRESIMS spectrum of tenellone G (**4**).

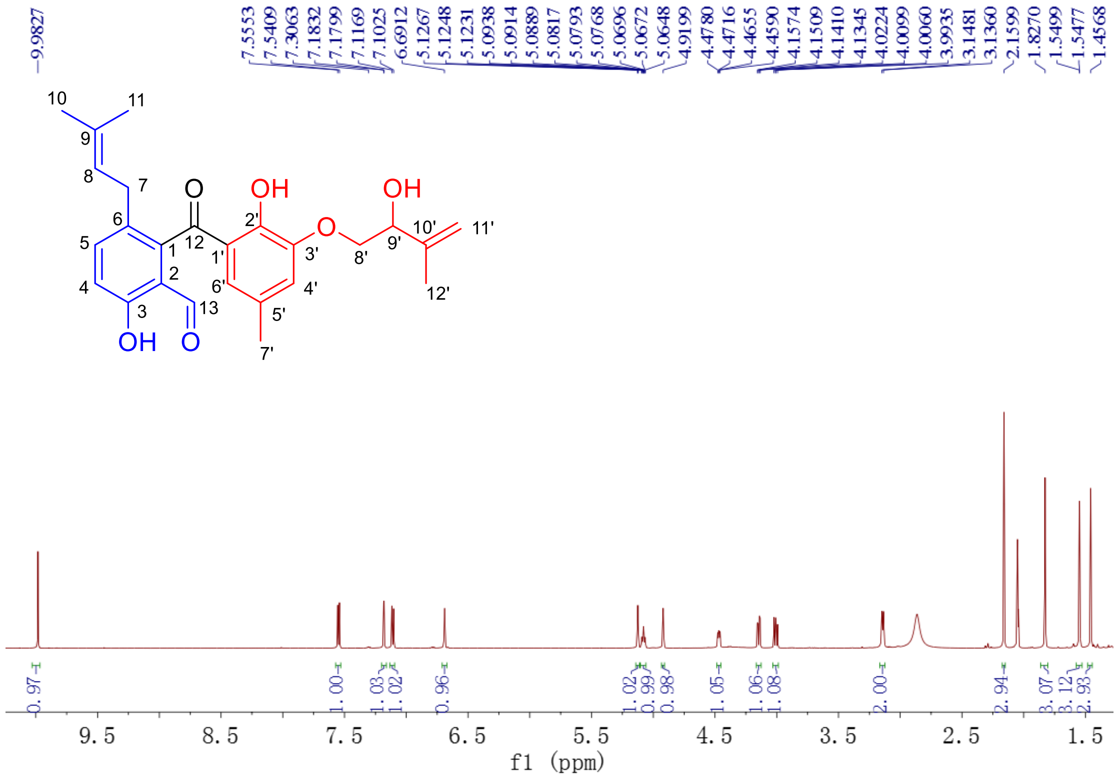


Figure S28. $^{1}$H NMR spectrum (600 MHz, $CD_3COCD_3$) of tenellone G (**4**).

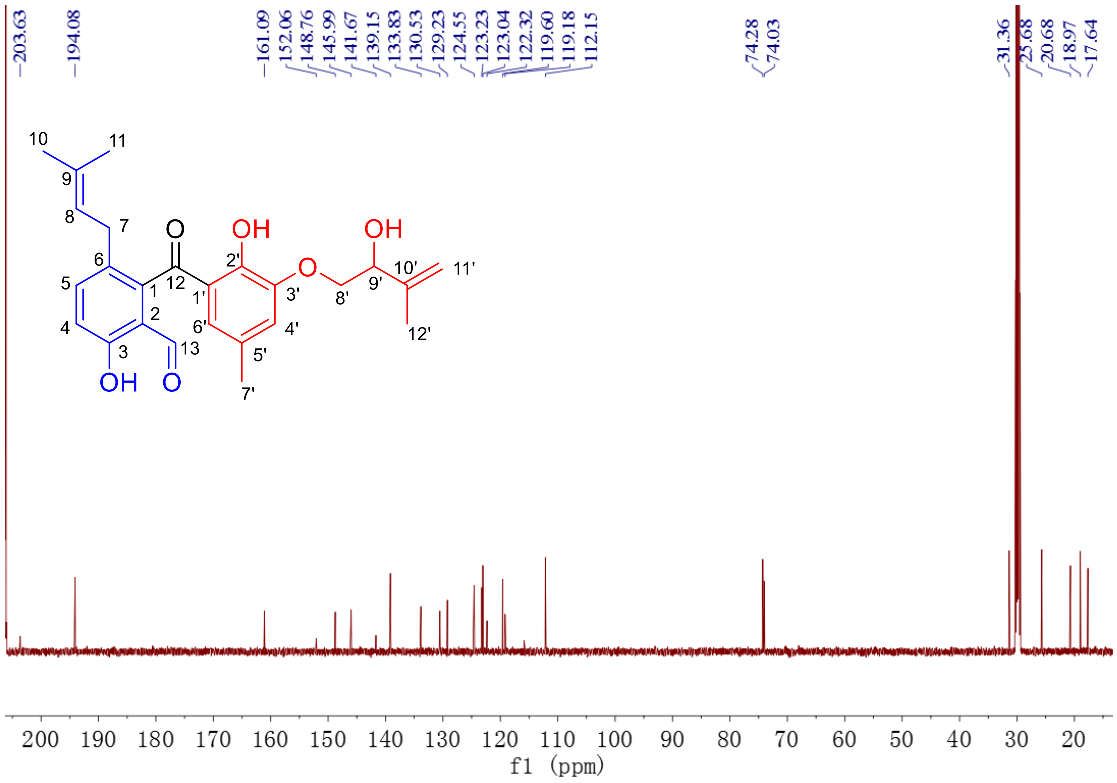


Figure S29. $^{13}$C NMR spectrum (150 MHz, $CD_3COCD_3$) of tenellone G (**4**).

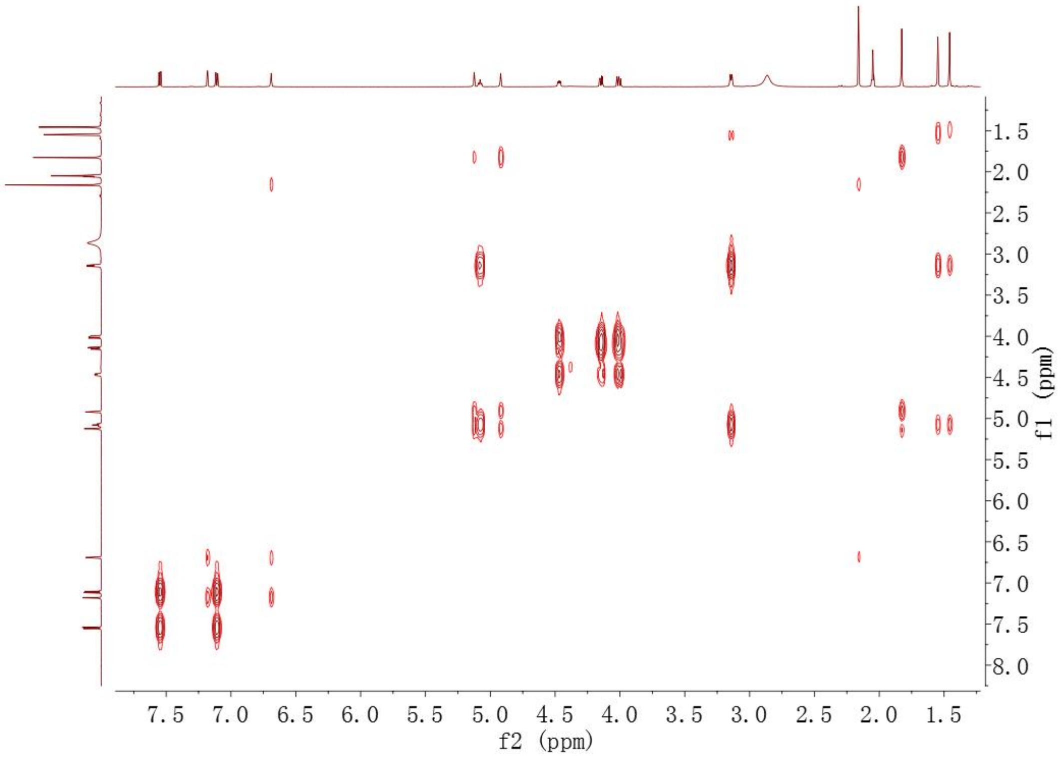


Figure S30. $^{1}$H-$^{1}$H COSY spectrum (600 MHz, $CD_3COCD_3$) of tenellone G (**4**).

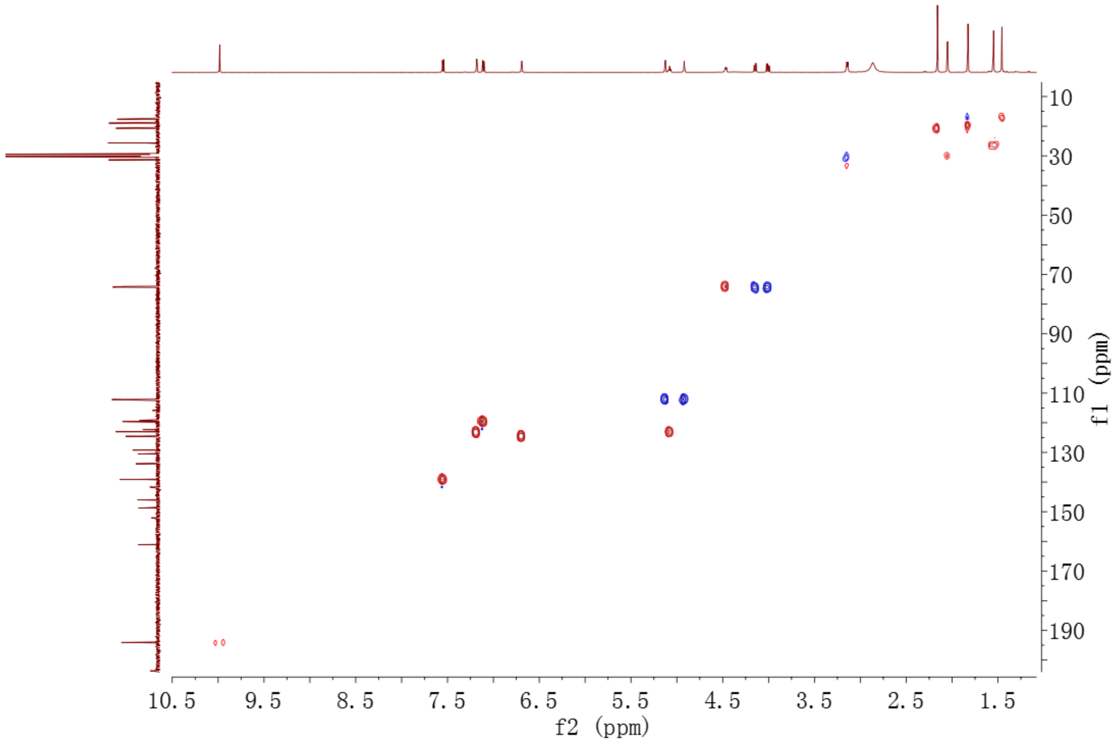


Figure S31. HSQC spectrum of tenellone G (**4**).

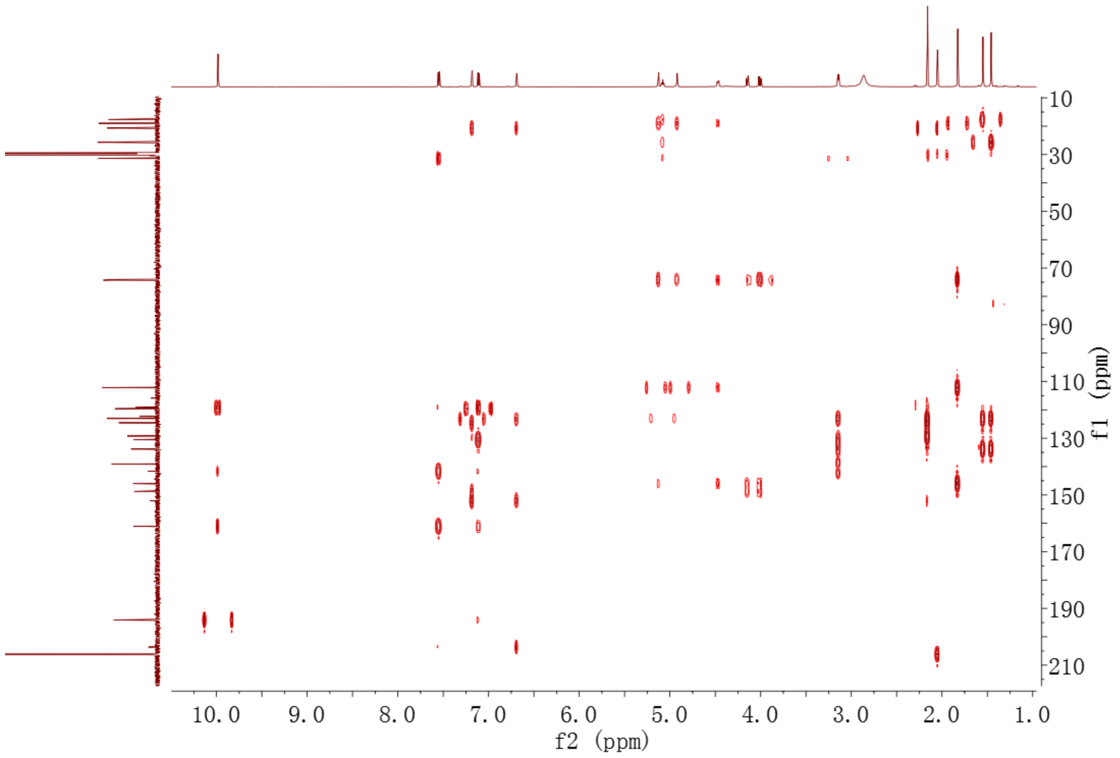


Figure S32. HMBC spectrum of tenellone G (**4**).

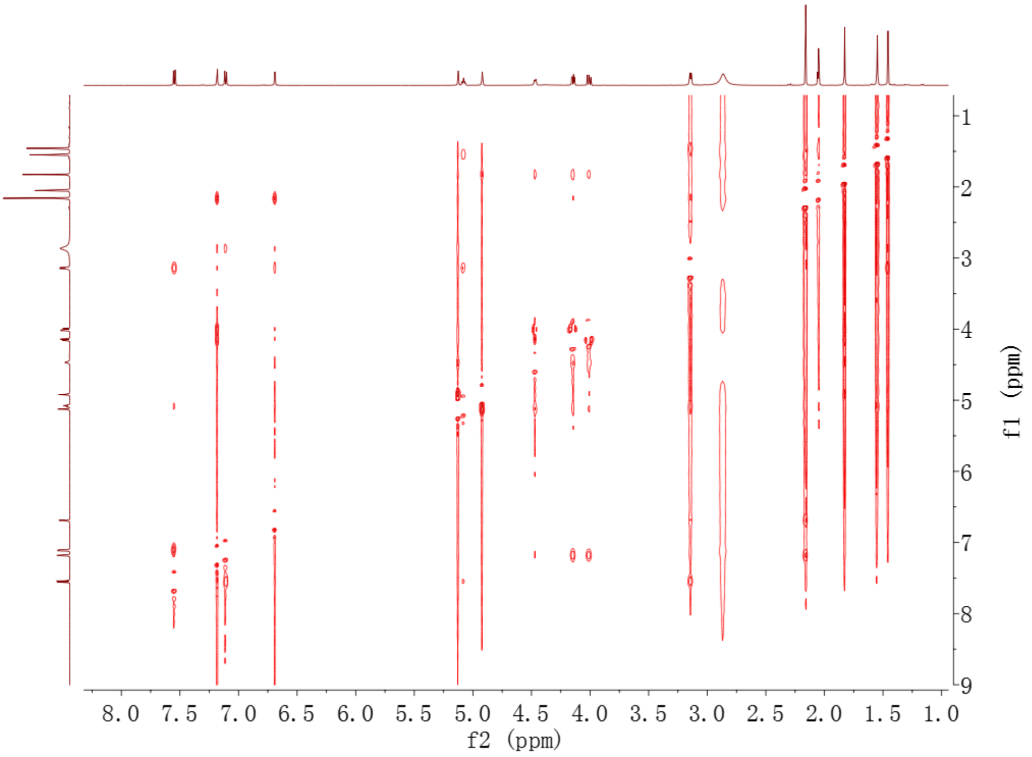


Figure S33. NOESY spectrum (600 MHz, $CD_3COCD_3$) of tenellone G (**4**).

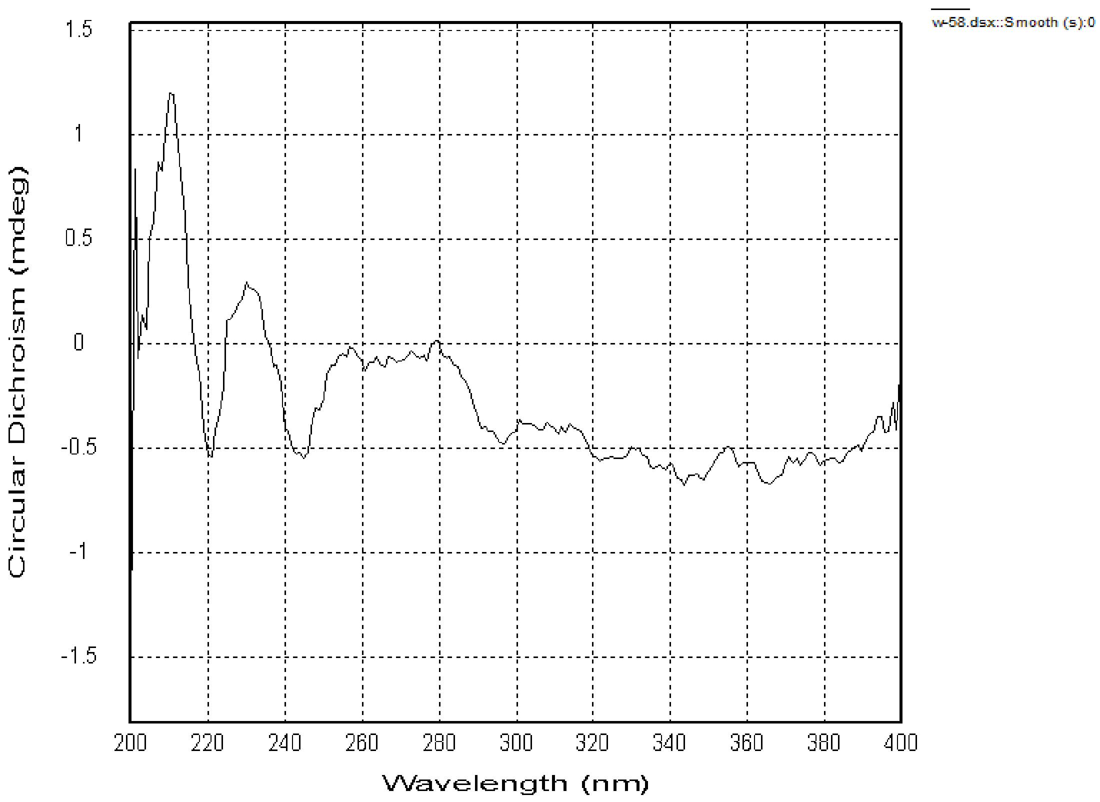


Figure S34. CD spectrum of tenellone G (**4**).

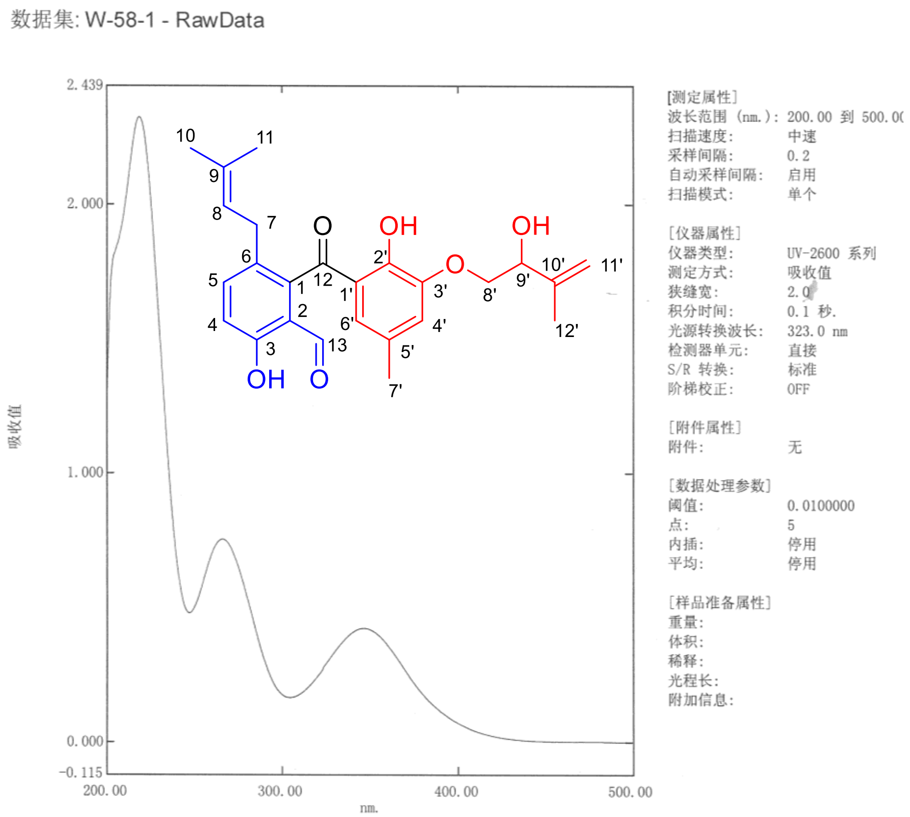


Figure S35. UV spectrum of tenellone G (**4**).

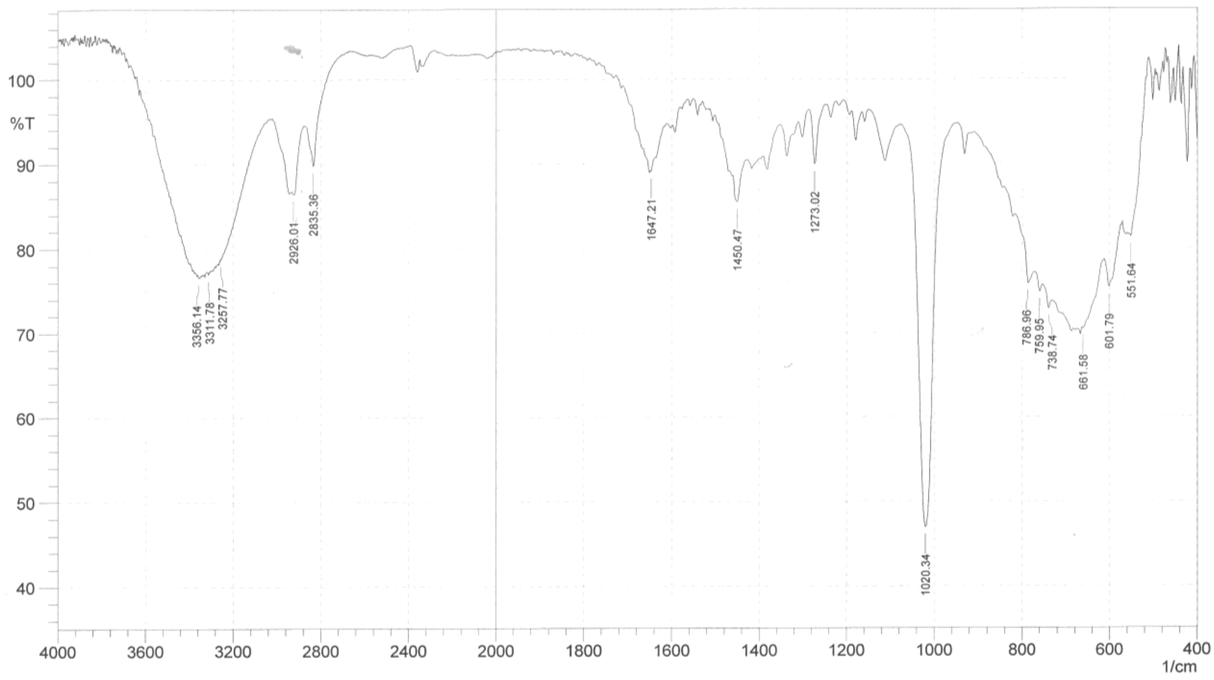


Figure S36. IR spectrum of tenellone G (**4**).

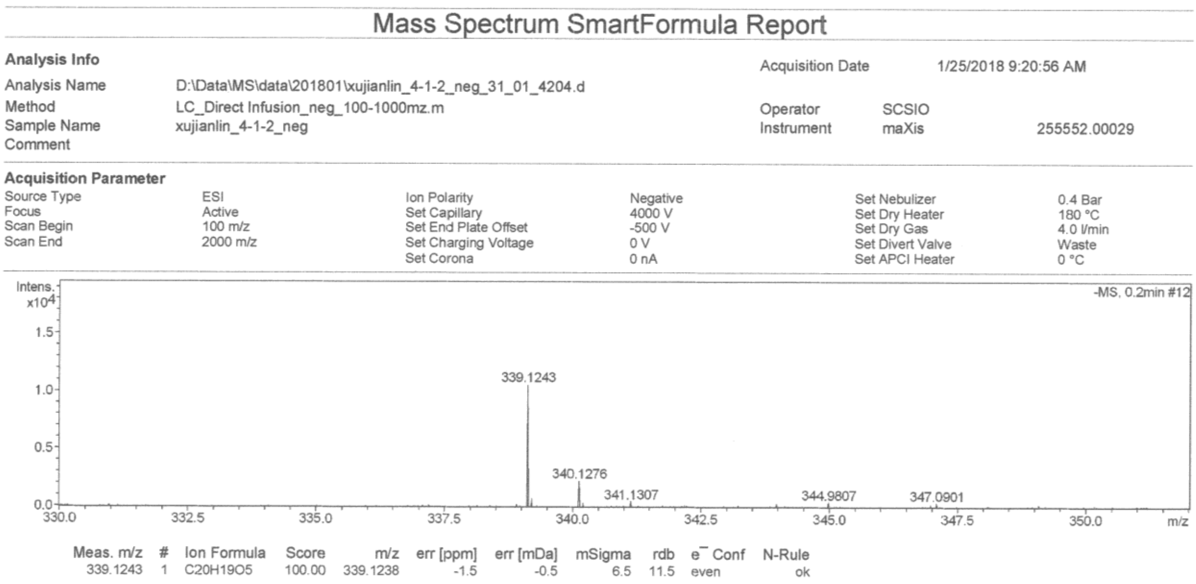


Figure S37. HRESIMS spectrum of tenellone H (**5**).

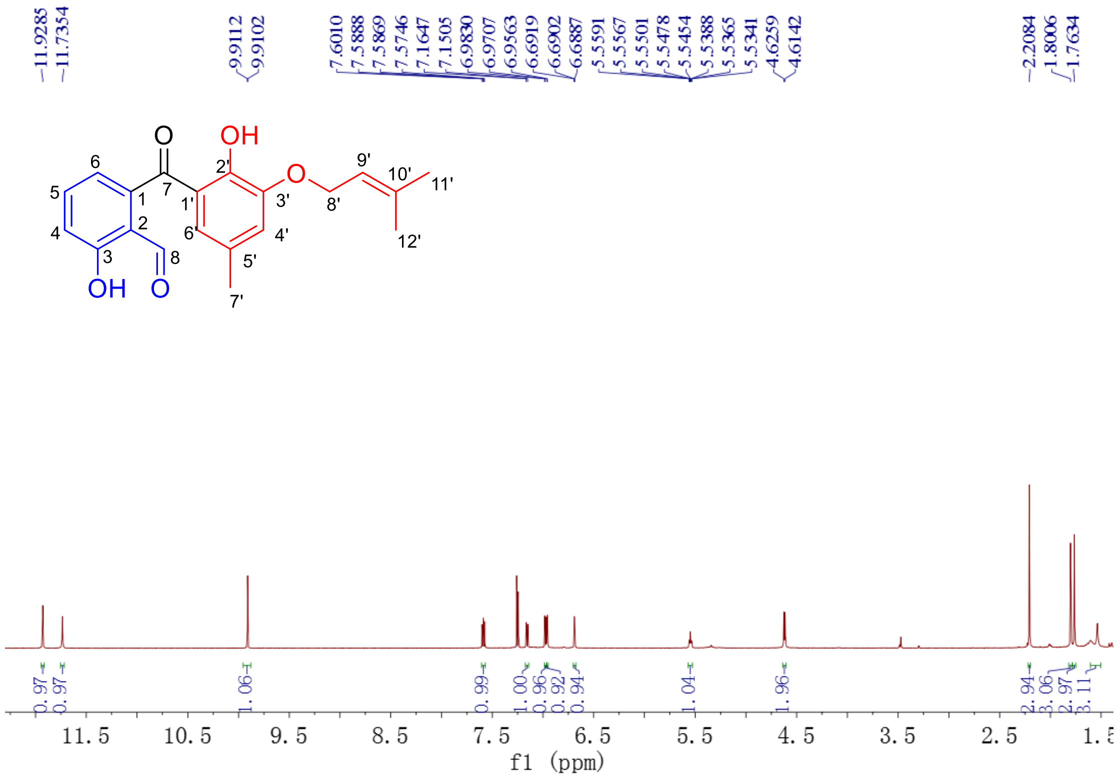


Figure S38. ¹H NMR spectrum (600 MHz, $CD_3Cl$) of tenellone H (**5**).

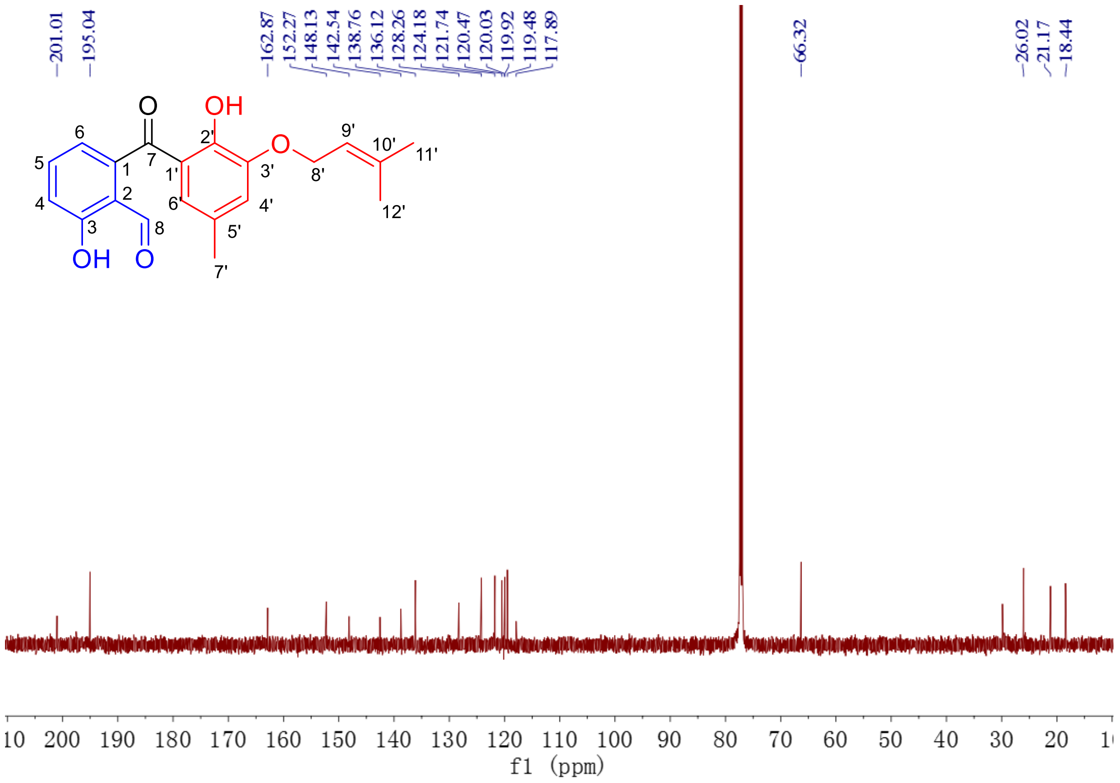


Figure S39. $^{13}$C NMR spectrum (150 MHz, $CD_3Cl$) of tenellone H (**5**).

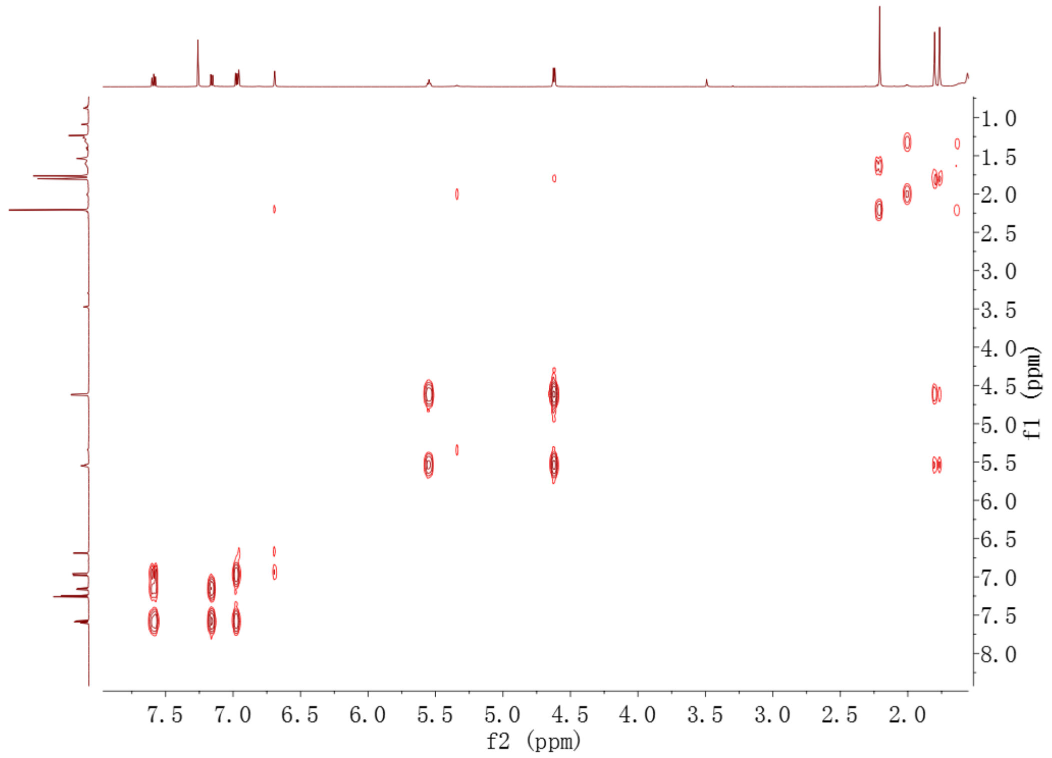


Figure S40. $^{1}$H-$^{1}$H COSY spectrum (600 MHz, $CD_3Cl$) of tenellone H (**5**).

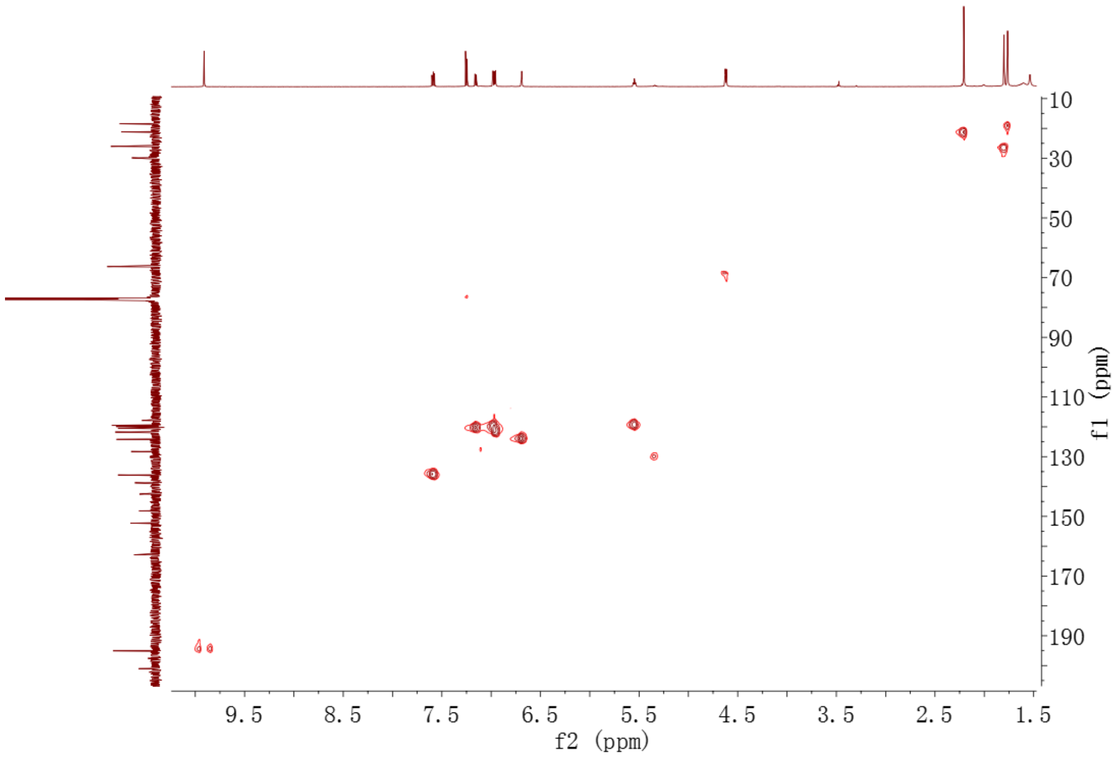


Figure S41. HSQC spectrum of tenellone H (**5**).

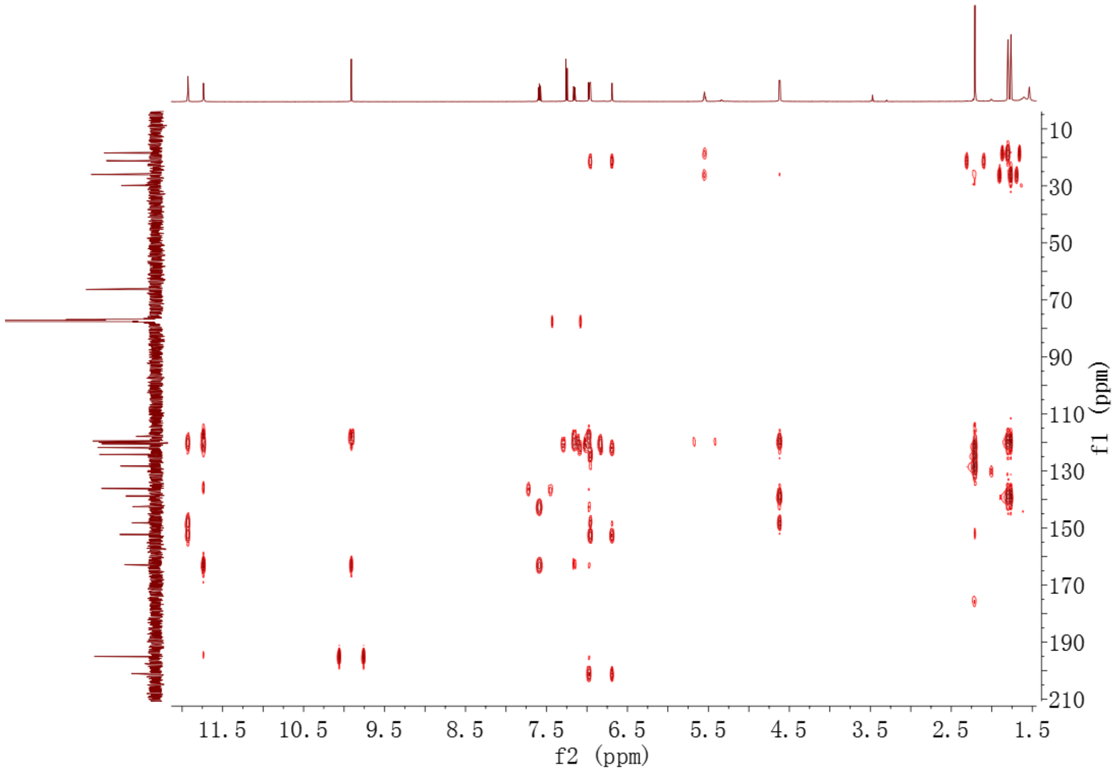


Figure S42. HMBC spectrum of tenellone H (**5**).

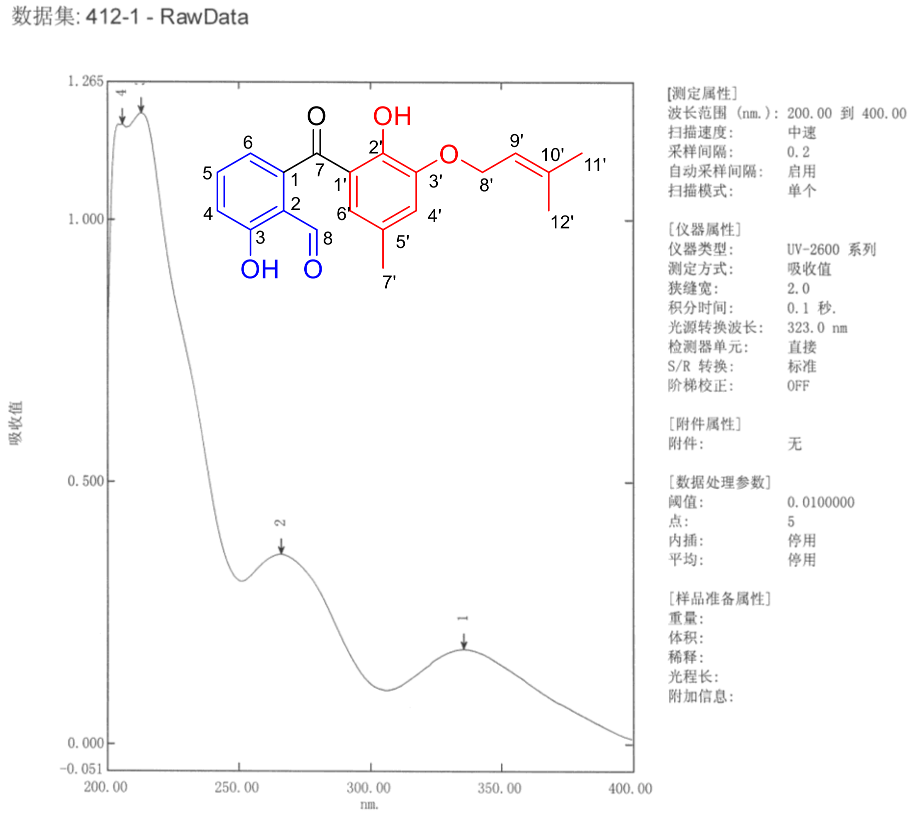


Figure S43. UV spectrum of tenellone H (**5**).

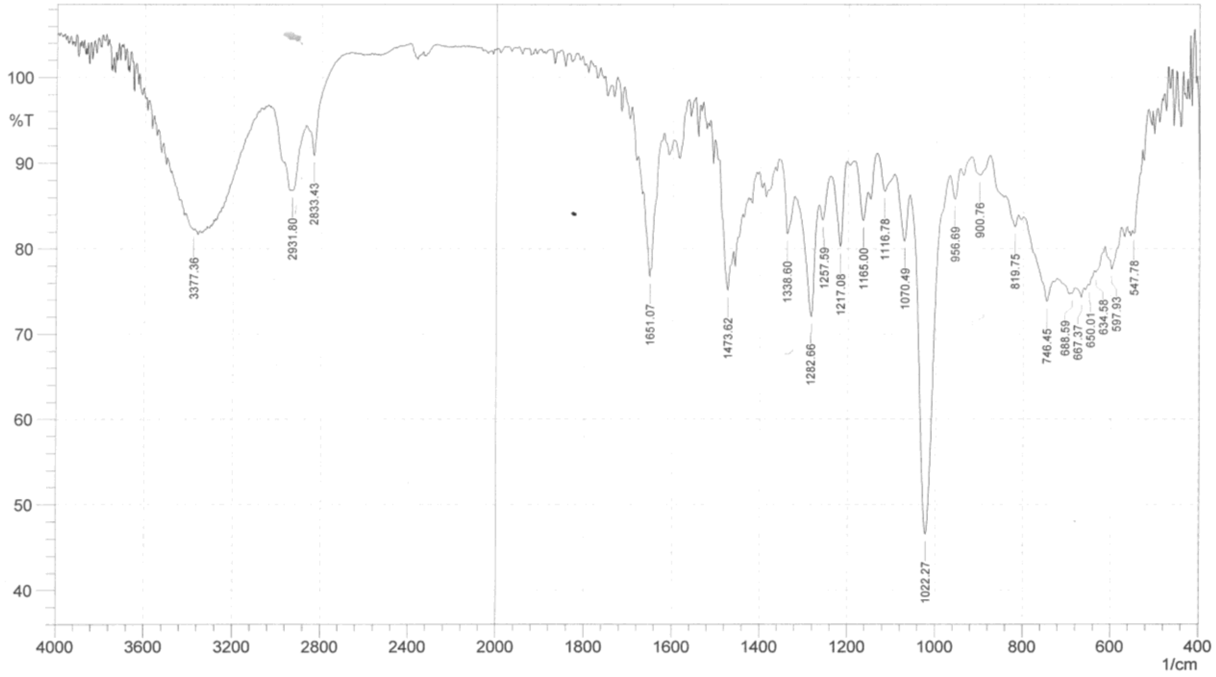


Figure S44. IR spectrum of tenellone H (**5**).

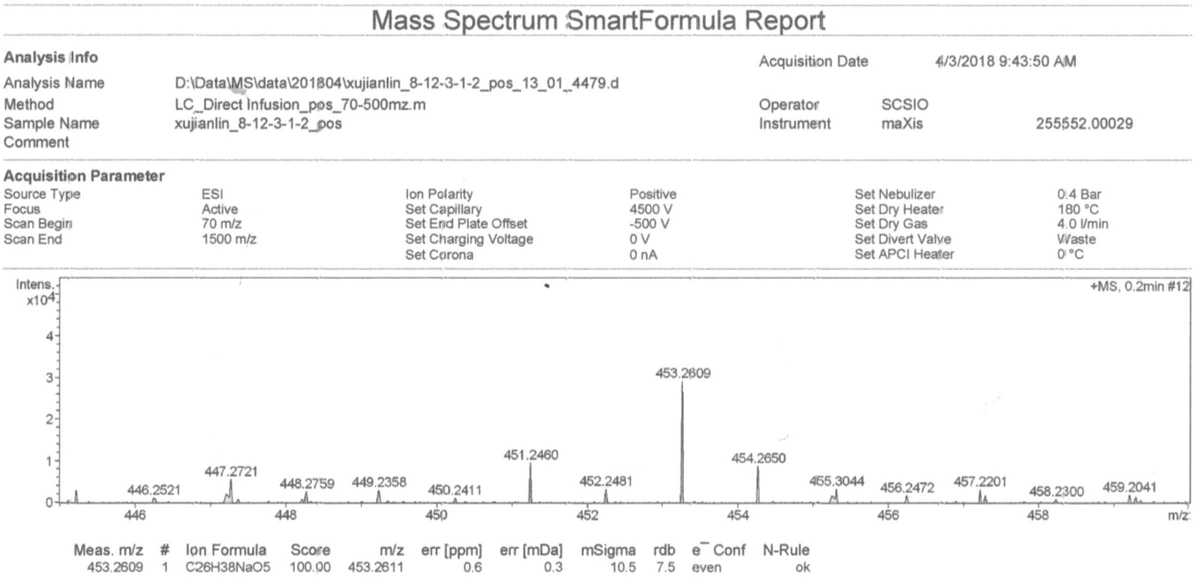


Figure S45. HRESIMS spectrum of lithocarin A (**7**).

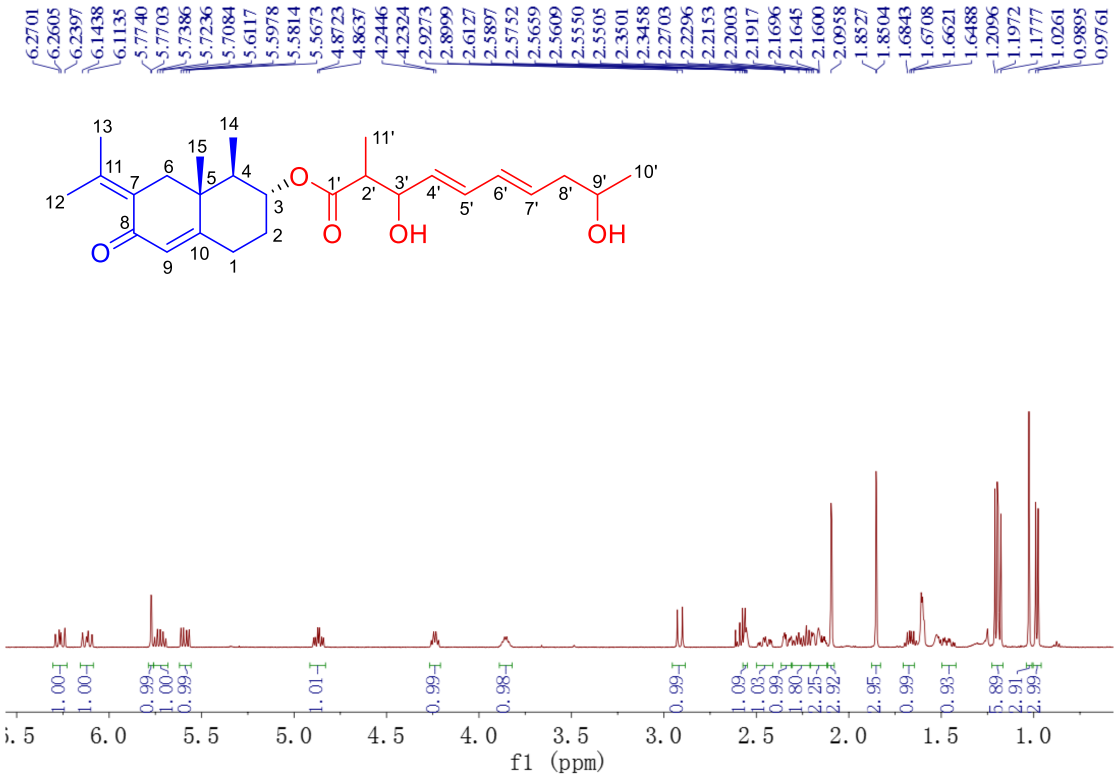


Figure S46. $^{1}$H NMR spectrum (500 MHz, $CD_3OD$) of lithocarin A (**7**).

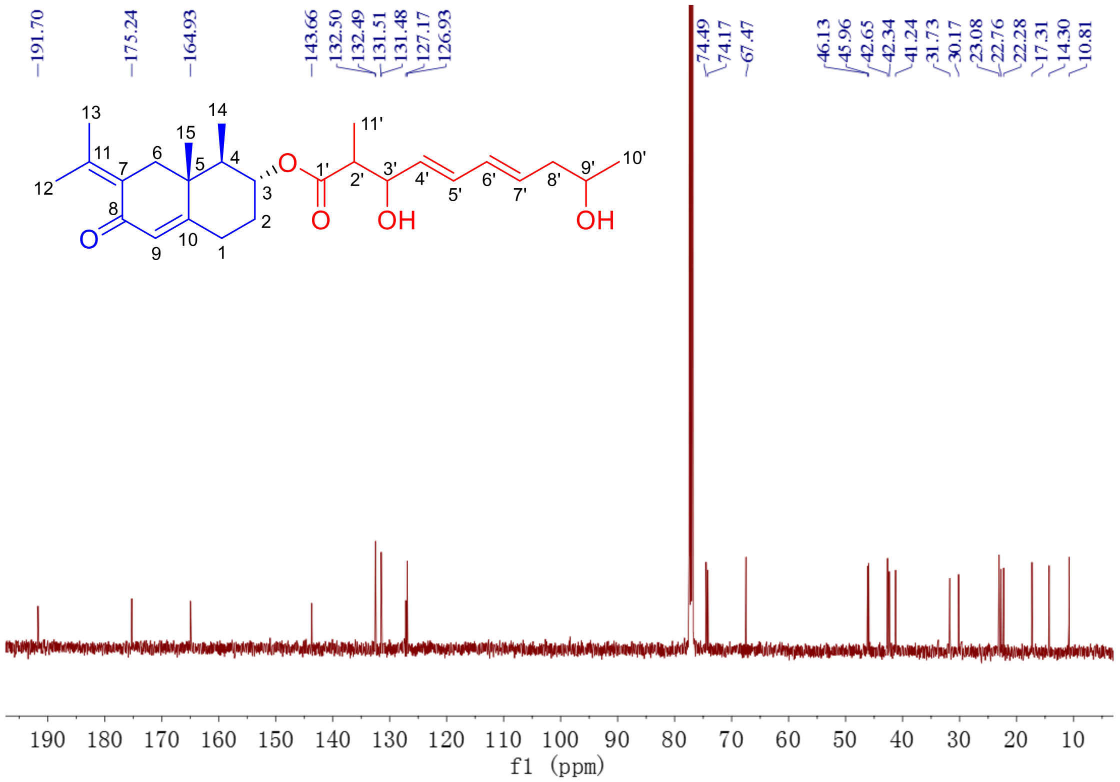


Figure S47. $^{13}$C NMR spectrum (125 MHz, $CD_3OD$) of lithocarin A (**7**).

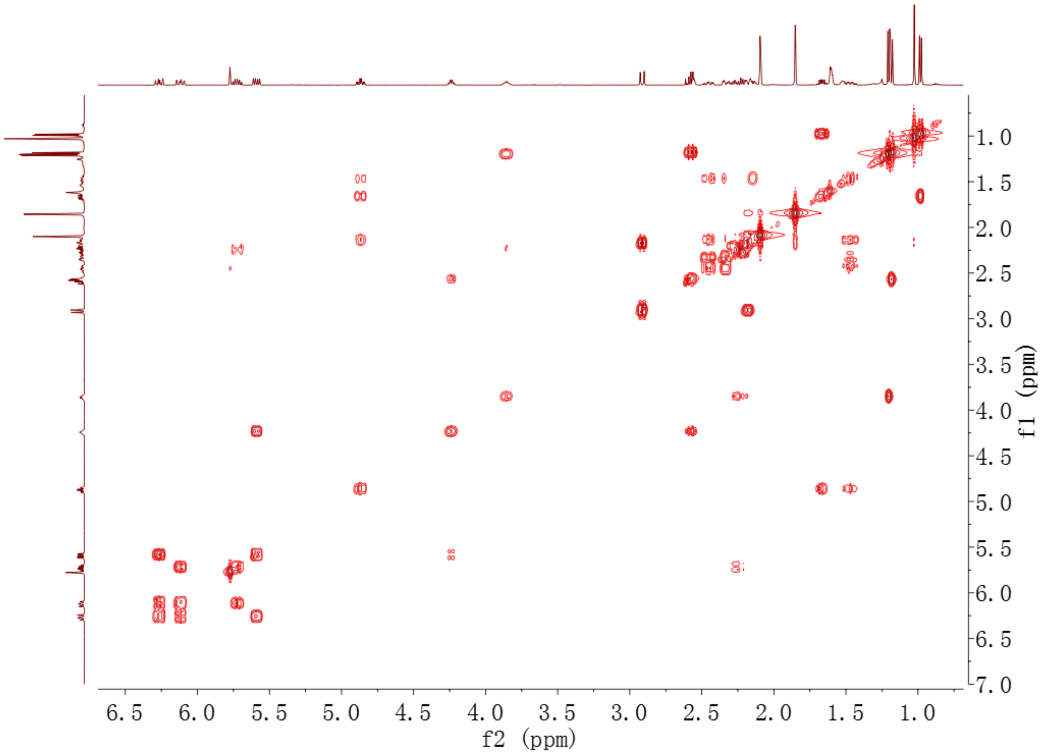


Figure S48. $^{1}$H-$^{1}$H COSY spectrum (500 MHz, $CD_3OD$) of lithocarin A (**7**).

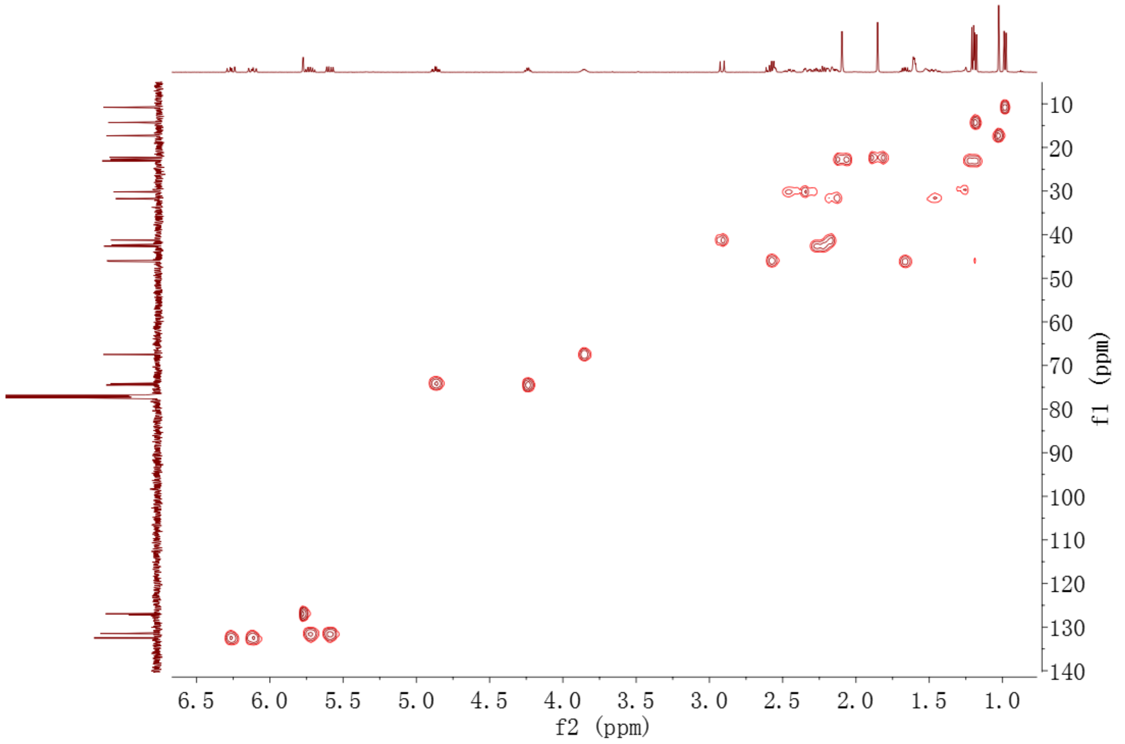


Figure S49. HSQC spectrum of lithocarin A (**7**).

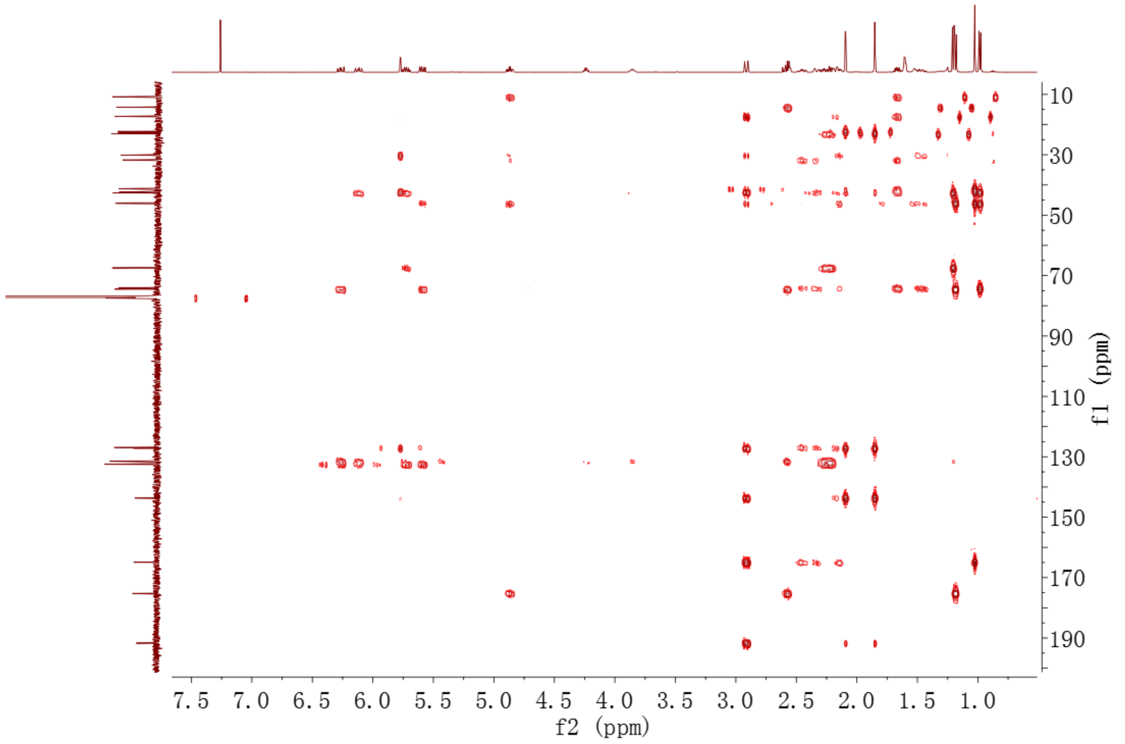


Figure S50. HMBC spectrum of lithocarin A (**7**).

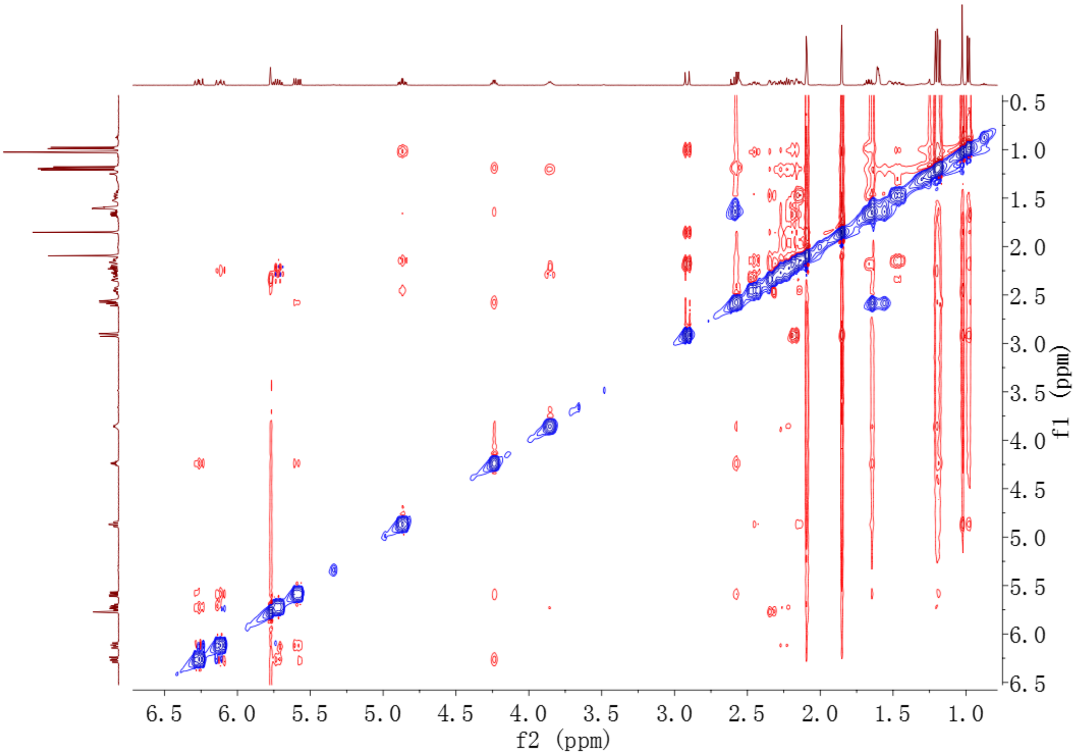


Figure S51. NOESY spectrum (500 MHz, $CD_3OD$) of lithocarin A (**7**).

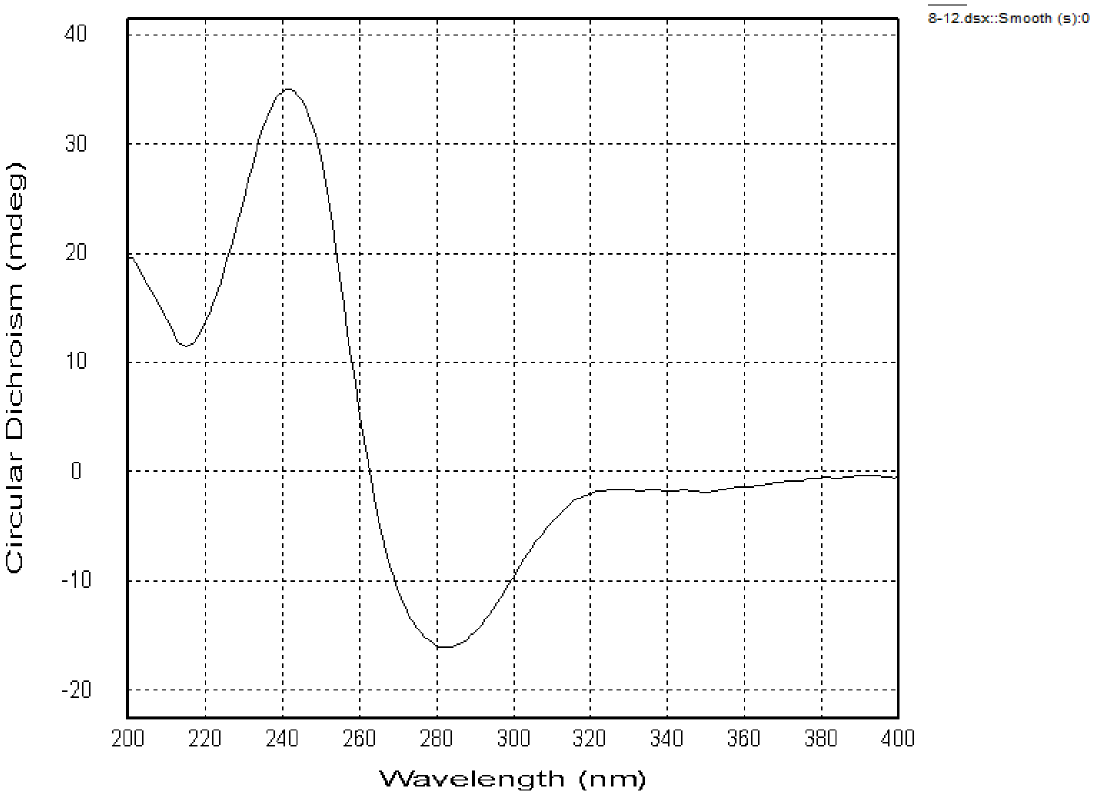


Figure S52. CD spectrum of lithocarin A (**7**).

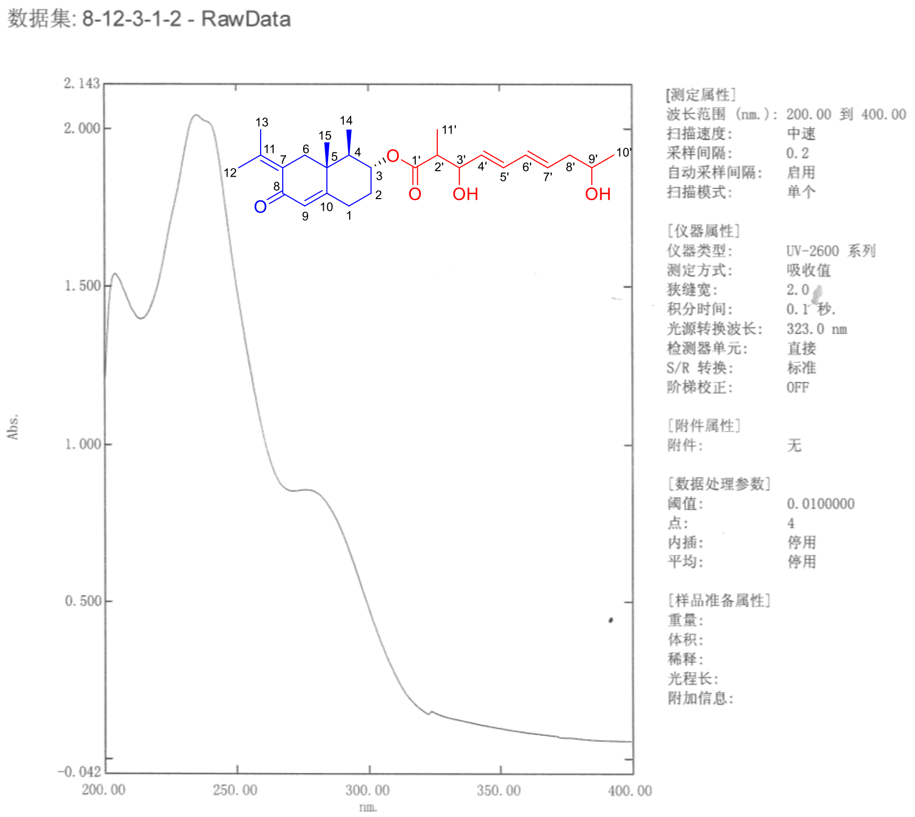


Figure S53. UV spectrum of lithocarin A (**7**).

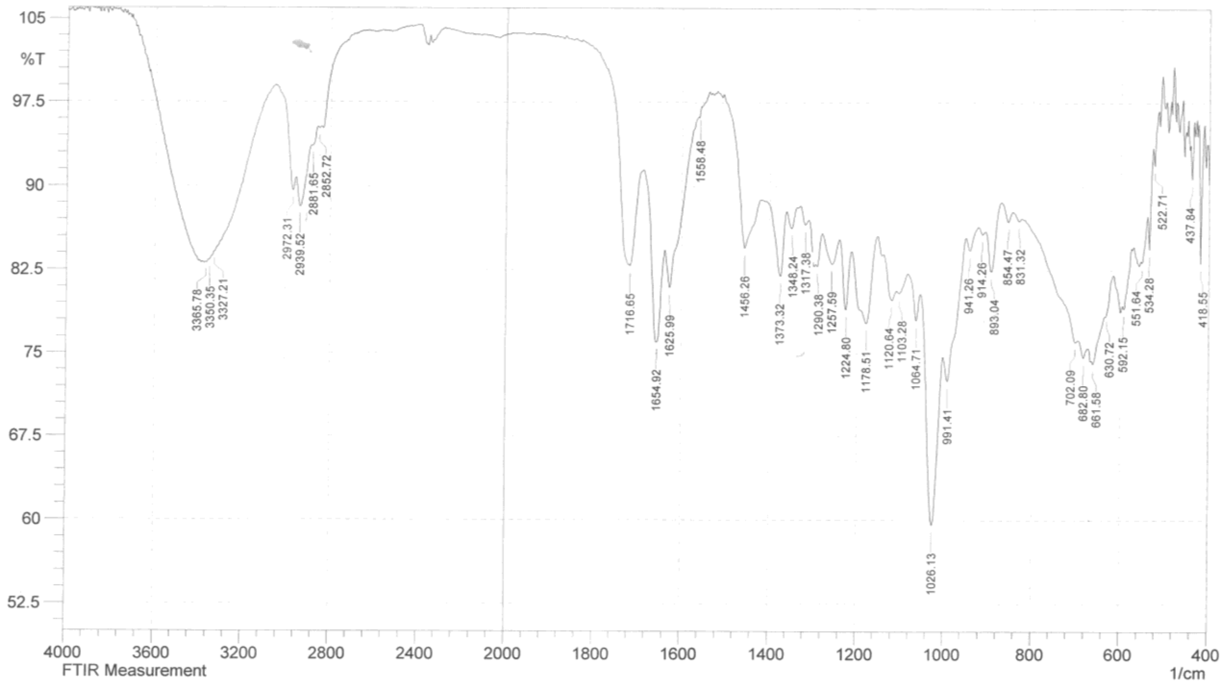


Figure S54. IR spectrum of lithocarin A (**7**).

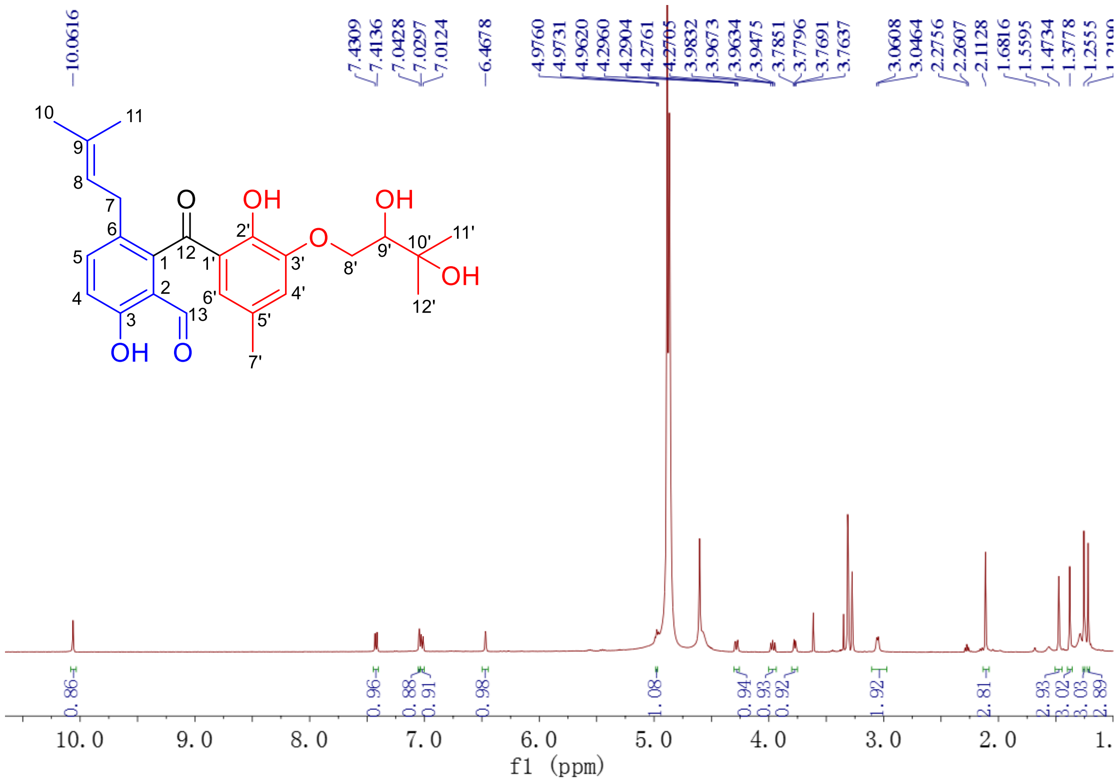

Figure S55. $^{1}$H NMR spectrum (500 MHz, CD3OD) of tenellone A (**6**).

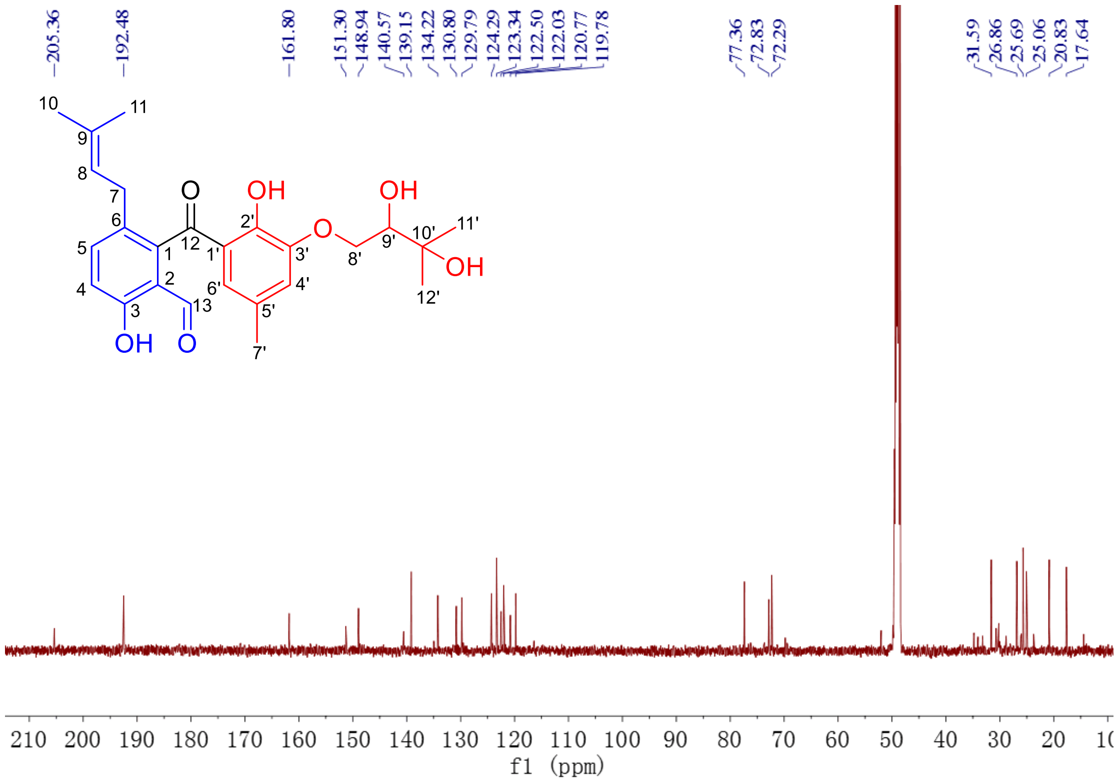

Figure S56. $^{13}$C NMR spectrum (125 MHz, CD3OD) of tenellone A (**6**).

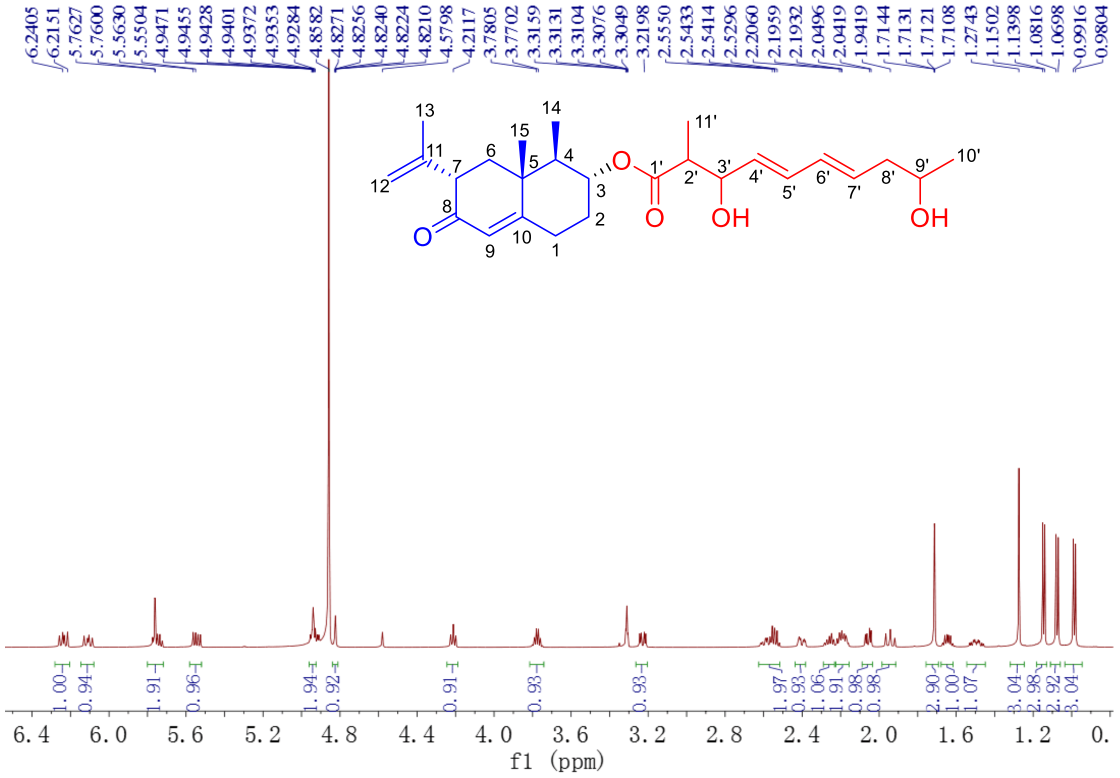

Figure S57. $^{1}$H NMR spectrum (500 MHz, $CD_3OD$) of AA03390 (**8**).

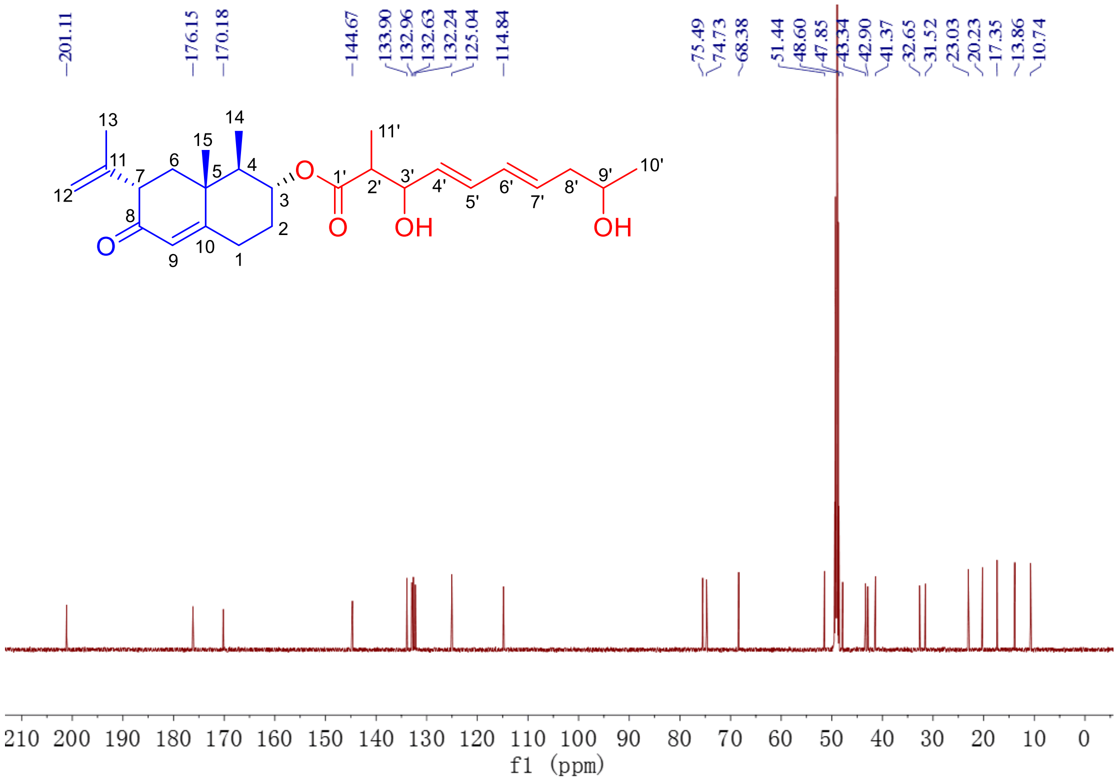

Figure S58. $^{13}$C NMR spectrum (125 MHz, $CD_3OD$) of AA03390 (**8**).
